# Evaluating health service outcomes of public involvement in health service design in high-income countries: a systematic review

**DOI:** 10.1186/s12913-021-06319-1

**Published:** 2021-04-20

**Authors:** Nicola Lloyd, Amanda Kenny, Nerida Hyett

**Affiliations:** Violet Vines Marshman Centre for Rural Health Research, La Trobe Rural Health School, La Trobe University, Bendigo, Australia

**Keywords:** Public involvement, Health service design, Evaluation, Outcomes, Systematic review, Co-design

## Abstract

**Background:**

Internationally, it is expected that health services will involve the public in health service design. Evaluation of public involvement has typically focused on the process and experiences for participants. Less is known about outcomes for health services. The aim of this systematic review was to a) identify and synthesise what is known about health service outcomes of public involvement and b) document how outcomes were evaluated.

**Methods:**

Searches were undertaken in MEDLINE, EMBASE, The Cochrane Library, PsycINFO, Web of Science, and CINAHL for studies that reported health service outcomes from public involvement in health service design. The review was limited to high-income countries and studies in English. Study quality was assessed using the Mixed Methods Appraisal Tool and critical appraisal guidelines for assessing the quality and impact of user involvement in health research. Content analysis was used to determine the outcomes of public involvement in health service design and how outcomes were evaluated.

**Results:**

A total of 93 articles were included. The majority were published in the last 5 years, were qualitative, and were located in the United Kingdom. A range of health service outcomes (discrete products, improvements to health services and system/policy level changes) were reported at various levels (service level, across services, and across organisations). However, evaluations of outcomes were reported in less than half of studies. In studies where outcomes were evaluated, a range of methods were used; most frequent were mixed methods. The quality of study design and reporting was inconsistent.

**Conclusion:**

When reporting public involvement in health service design authors outline a range of outcomes for health services, but it is challenging to determine the extent of outcomes due to inadequate descriptions of study design and poor reporting. There is an urgent need for evaluations, including longitudinal study designs and cost-benefit analyses, to fully understand outcomes from public involvement in health service design.

**Supplementary Information:**

The online version contains supplementary material available at 10.1186/s12913-021-06319-1.

## Background

Internationally, there is an expectation that health services will involve the public in health service design. The assumption is that public involvement results in services that improve health and quality of life [1–6]. While potential outcomes of public involvement are widely discussed, these are primarily focused on outcomes for participants. Less is known about the outcomes of public involvement for health services or how these have been evaluated [4, 7–10]. This review addresses this gap.

The term ‘outcome’ is used in literature but rarely defined. For this review, any proposed or eventuating positive or negative change to the health service, or product produced, was considered an outcome. Public involvement in health services is promoted as an ethical and democratic right [11, 12], and valuable regardless of outcomes [13]. However, in an era of finite health service resources, robust evidence of the outcomes of public involvement is critical for health service planning [14]. Public involvement requires investment of time and energy for the service and public participants. Financial and other costs can be significant [12]. Participants want their contributions to make a difference, and services want good value for the resources required [15, 16]. Without robust evaluation of outcomes, there is a risk that public involvement is merely a tokenistic attempt to comply with government policy and accreditation standards [17, 18].

Systematic reviews to this point have explored patient participation in shared decision making [19], public involvement in health services in the United Kingdom [20], and the acute healthcare setting [21]; while another is somewhat dated [7]. In a recent review, Boivin et al. explored evaluation tools for patient and public engagement in research and health system decision making [9]. While they found 27 patient and public engagement evaluation tools, most lacked in scientific rigour and conceptual underpinnings, and most focused on evaluations of the engagement process and context rather than outcomes [9]. Comprehensive, purposeful evaluation is needed to understand what difference public involvement makes to health services and the broader community. Given international interest in public participation, a review of outcomes and evaluation approaches is overdue.

The aim of this systematic review was to a) identify and synthesise what is known about health service outcomes of public involvement b) document how outcomes were evaluated. The review questions were: What are the outcomes for health services of public involvement in health service design? Have these outcomes been evaluated, and if so, how?

## Methods

The review protocol was registered on the International Prospective Register of Systematic Reviews (PROSPERO) (https://www.crd.york.ac.uk/prospero/, registration number CRD4201809237). The review is reported according to the PRISMA statement.

### Definitions used in this review

There is a lack of consistency in defining and conceptualising public involvement, and many overlapping terms exist [22]. Research on public involvement is documented from a wide variety of disciplines and settings and has emerged from disparate policies, contexts and social movements [23]. In this review, public involvement is defined as any interaction between members of the public and employees of a health service, government, or other organisation, with the agreed purpose of designing or re-designing health services. Public involvement was chosen as an umbrella term in the absence of a gold standard. For the purpose of this review user involvement, patient and public involvement, consumer engagement, public participation, community participation, co-design, co-production, or a combination of these terms were accepted.

The definition of a health service was guided by the Australian Privacy Act of 1998 [24], which in summary includes any activity assessing, maintaining, improving, managing, or recording an individual’s health; diagnosing or treating illness, disability, or injury; or dispensing prescribed drugs or medicinal preparations by a pharmacist. Health service design (or re-design) was deemed any activity which proposed or implemented a change to improve service quality [25]. This included developing new models, priority setting, quality improvement projects, and physical environment planning, if they occurred in the context of health service design.

### Search strategy

Breadth in study type was deemed necessary to ensure a comprehensive exploration and synthesis of all relevant studies. Searches were completed in March 2018 in MEDLINE, EMBASE, the Cochrane Library, PsycINFO, Web of Science, and CINAHL, and updated in December 2019. There were no date restrictions. A preliminary search of two databases was used to identify additional keywords, index terms or subject headings. The final search strategy was developed in consultation with a university librarian and included combining terms from each of the following groups: health service, involvement, public and service design (see Additional file 1 for example of MEDLINE search strategy).

The search was augmented by hand-searching references lists of included articles, and forward-searching for relevant articles where there was reference to plans for further implementation or evaluation studies.

### Eligibility criteria

Articles were included if they were original research published in academic peer-reviewed journals with evidence of public involvement in health service design or re-design, with reported health service outcomes. Outcomes for participating individuals or evaluations of how public involvement was conducted were not the focus of this review. Due to differences in health service design and delivery between low, middle, and high-income countries, the review was limited to high-income countries, defined as members of the Organisation for Economic Co-operation and Development (OECD) at the time the search was conducted [26]. There were no date restrictions. Articles were excluded if they were unavailable in the English language due to time and cost limitations.

### Article selection

The systematic review was managed with Covidence™ [27]. Titles and abstracts were reviewed for eligibility independently by the first author and a second member of the research team. Any disagreements were resolved by discussion. The same screening and decision process were used for full-text article eligibility.

### Data extraction

The first author extracted data from each article using a standardized Excel spreadsheet. The form was piloted on 10% of randomly selected articles, and modifications were made following discussion from all three researchers. Data extraction included the following: first author; year of publication; study design; level of involvement using the International Association for Public Participation (IAP2) framework [28]; study aim/s and objective/s; details of health service; country; definition/conceptualisation of public involvement; public recruitment procedures; characteristics of public participants; data collection and analysis methods; reported outcomes; study conclusions; strengths and limitations; and details of evaluation including any frameworks or tools. Data were extracted by the first author and reviewed by a second author. Discrepancies were compared against the original article, and disagreements were resolved by discussion.

The IAP2 [28] is a spectrum of five levels of public participation, ranging from the lowest level ‘Inform’, to the highest level ‘Empower’. The level of public involvement in each study was rated on this spectrum. Where projects had multiple components involving the public at different levels or several publications relating to the same study, the highest overall level of public involvement was recorded.

### Quality assessment

Quality appraisal of studies was assessed using the Mixed Methods Appraisal Tool (MMAT, 2018 Version) [29]. The MMAT was designed for appraising studies of various designs, has been pilot tested for efficiency, content validity, and reliability, and has been used in over 100 systematic reviews [30, 31]. Each criterion is assessed as either met (‘Yes’), not met (‘No’) or insufficient information for judgement (‘Can’t tell’). The publicly available MMAT Excel spreadsheet was used to record criterion assessment and reviewer comments [32]. The critical appraisal guidelines for assessing the quality and impact of user involvement in health research were used [33]. These guidelines consider the quality of research relating to public involvement. Each article was assessed for compliance against the nine criteria. A scoring system of one point per criterion met was used to assess the overall quality of included articles. Common themes or areas of concern regarding quality were identified to support recommendations for improving reporting or rigour in future studies [34]. Quality assessment was completed by the first author and confirmed by a second author.

### Data synthesis

Content analysis, as described by Hsieh and Shannon [35] was used for data synthesis. This inductive approach involved familiarisation with included articles, then exploration and defining of the emerging categories and themes relevant to the research questions. Tabulation and frequencies of the categories derived from the data enabled exploration of various factors, such as the type of outcomes described and the level they occurred at [7, 36] Additional file 2.

## Results

### Included studies

**Fig. 1:**
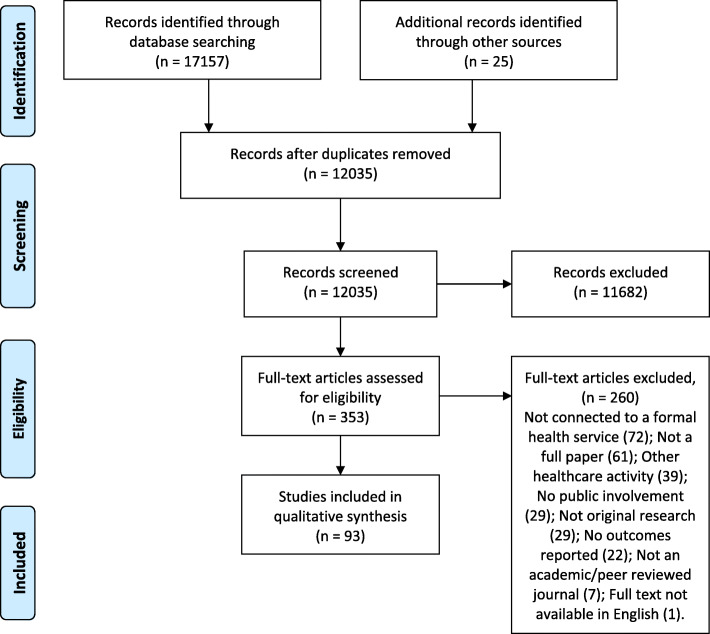
Systematic review process flowchart

The study selection process is outlined in Fig. 1. A total of 93 articles were included in this review. There were 90 original public involvement studies and the outcomes for these are reported together in Additional file 2.

### Study characteristics

Publication dates of included articles ranged from 1994 to 2019, with 61.4% of articles published in the last 5 years. Of the 93 included articles, 55 were qualitative (59.1%); 34 were mixed methods (36.6%); and 4 were quantitative (4.3%). The majority of articles originated in the United Kingdom (*n* = 49, 52.7%), followed by the United States of America (*n* = 12, 12.9%), Australia (*n* = 11, 11.8%), and Canada (*n* = 10, 10.75%). The remainder were from Norway (*n* = 4, 4.3%), Spain (*n* = 2, 2.1%), Sweden (*n* = 2, 2.1%), Denmark (*n* = 1, 1.1%), New Zealand (*n* = 1, 1.1%), and South Korea (*n* = 1, 1.1%). The most common service type was mental health (*n* = 23, 25.6%), followed by multiple/all (*n* = 11, 12.2%), chronic/complex needs (*n* = 7, 7.8%), oncology (*n* = 6, 6.7%) and women’s health (*n* = 5, 5.6%). Authors commonly reported that studies were conducted across multiple settings in a health service (*n* = 24, 26.7%), followed by outpatients (*n* = 18, 20.0%), community (*n* = 17, 18.9%), primary care (*n* = 13, 14.4%) and acute inpatients (*n* = 12, 13.3%).

The articles varied in the detail that was provided regarding definition, rationale, or context for the concept of public involvement. For example, formal definitions of key concepts were given in some articles (e.g., of co-production [37]), while others provided an overview of the history and usage of the involvement activity (e.g. Experience based co-design [38], or presented arguments for how and why the public should be involved (e.g. [39]). There were no definitions or conceptualisation of public involvement in almost half of articles (*n* = 43, 46.2%) [40–82]. Where reported, public participant numbers ranged from 4 [83], to an estimated 1200 [84], though no numbers were given in 13 articles (14.0%) [45, 47, 49, 58, 69, 72–74, 77, 85–88], and incomplete or estimated numbers were provided in a further 25 (26.9%) [40, 43, 44, 50, 51, 56, 57, 61–63, 66, 76, 82, 84, 89–99]. Some authors provided demographic data of public participants, such as age, gender, medical condition and/or race, however, there was no detail provided in more than half of articles (*n* = 52, 55.9%) [37, 38, 40, 41, 43–51, 55, 58, 59, 61, 63, 66, 68, 69, 71–74, 77, 79, 81–88, 93, 95–110]. There were no studies where IAP2 public involvement level was rated as ‘Inform’, or ‘Empower’. Of the included 90 studies, 37 (41.1%) were rated as ‘Consult’, 29 (32.2%) as ‘Involve’, and 24 (26.7%) as ‘Collaborate’ (Additional file 2).

### Review question 1: what are the health service outcomes of public involvement in health service design?

The definition of an outcome for this review was intentionally broad, being any proposed or eventuating positive or negative change to the health service or product produced. Table 1 documents the range of health service outcomes that were reported. Outcomes have been grouped into categories of *discrete products* [130], *improvements to health services*, and *system or policy level change/s*. In some articles, multiple types of outcomes were described, and these were categorised as the highest-level outcome reported. Outcomes were produced at the service level (*n* = 46, 51.1%), across services (*n* = 16, 17.8%), and across organisations (*n* = 28, 31.1%). Full detail of the types and range of health service outcomes are outlined in Additional file 2.

**Table 1:** Health service outcomes

Type of outcome	Studies
*Discrete products (n = 32, 36.5%)*	
Design or redesign of patient or program information products	[54, 56, 66, 67, 73, 86, 91, 98, 100, 101, 105, 107, 109, 111–117]
Patient decision aids	[50, 76, 82]
Frameworks, indicators or measures to guide or evaluate services	[39, 40, 55, 75, 78, 116, 118, 119]
e-health products for managing health conditions, peer support, and/or planning healthcare visits	[42, 43, 45, 53, 57, 58, 60, 74, 80, 81, 89, 92, 94, 97, 102, 108, 120–124]
e-learning training packages for staff	[83]
*Improvements to health services (n = 39, 43.%)*	
New education, support, recreational, or clinical programs, or new clinics or services	[47, 56, 64, 69, 84, 92, 93, 96, 99, 100, 109, 110, 112, 113, 116, 125]
Improvements to enhance patient experience e.g., physical environment, menu, availability of personal belongings, service accessibility, and improving trust, communication and decision-making processes with healthcare professionals	[38, 54, 68, 77, 78, 86, 87, 90–92, 96, 100, 101, 106, 107, 109, 111, 115–117, 125, 126]
Patient-oriented health records, consultant summary letters or discharge summaries (and processes to support their use in the service)	[61, 62, 70]
Improvements to service processes, e.g., record keeping, data sharing, medication dispensing, care pathways, and appointment and recall systems	[38, 46, 54, 65, 68, 77, 79, 88, 90, 101, 106, 107, 109, 114, 125, 126]
Processes for eliciting patient and family feedback	[105, 116]
Redesign and/or expansion of existing services	[48, 104, 127]
New volunteer or staff positions	[38, 44, 98, 117, 127]
Design of staff recruitment processes, induction, education and training	[37, 38, 48, 54, 68, 87, 100, 109, 112, 117, 125]
*System or policy level change/s (n = 19, 21.1%)*	
Strategies to reconfigure or further develop services	[41, 51, 52, 96]
Development of a conceptual model of recovery	[128]
Prioritisation process to develop action plan for local service provision	[103, 113]
Formation of local health action groups, steering groups, or committees	[84, 93]
Developing infrastructure for patient and family engagement in decision-making processes	[95]
Spread of experience-based co-design processes to other services and organisations	[38]
Development of a quality assurance model for evaluating service improvements or frameworks to measure or evaluate service quality	[126, 129]

### Review question 2: have these outcomes been evaluated, and if so, how?

Evaluation of the reported outcome was outlined in less than half of studies (*n* = 39, 43.3%) [38, 41–45, 47–50, 53, 54, 56–59, 61, 65, 66, 68–71, 80, 81, 83–85, 87, 92, 93, 99, 102, 105, 107, 109, 110, 123, 131] (Additional file 2). Some evaluations were reported additionally [59, 70, 107] or exclusively [50, 53, 61, 66, 71, 76, 81, 83, 85, 102, 123, 131] in separate publications. We found examples of mixed methods (*n* = 21, 53.8% of projects which completed evaluation), quantitative (*n* = 10, 25.6%), and qualitative outcome evaluations (*n* = 8, 20.5%). (Additional file 2).

#### Evaluation method

We observed some trends regarding type of outcome and evaluation method chosen. Studies that resulted in discrete products (e.g., information leaflets, decision tools) were typically evaluated using pre-post measures of knowledge and/or health literacy [48, 50, 61]. For outcomes that included apps and e-health interventions, usability [42, 80, 132–134], acceptance [45], feasibility [57, 135–137], and perceived confidence in self-management [102] were measured. Studies evaluated over longer periods explored health outcomes using, for example, laboratory tests, neonatal outcomes, and body mass index with comparisons to prior to the new service or intervention, or in trials that compared an intervention to a control group.

#### Qualitative evaluation

Qualitative evaluation via participant feedback was common. Data were collected through interviews [38, 42, 44, 45, 58, 59, 62, 65, 72, 80, 81, 84, 87, 93, 100, 105, 109, 123, 138], focus groups [44, 45, 62, 70, 136], questionnaires or surveys [42, 45, 48, 59, 62, 66, 84, 100], evaluation forms [92], online collection of comments [70], and a yarning circle, “..a culturally-appropriate form of group discussion …” ([93], p.5). In one study, it was noted that “ …researchers spoke with consumers and other advocates…to elicit their opinions about how the public mental health system has changed” ([49], p.46). Stakeholder views were rarely measured using standardized tools, with the exception of the study by Cook et al. [85], which used the Client Services Questionnaire in a subsequent randomized controlled trial [139].

#### Quantitative evaluation

Data on indicators such as service usage, treatment uptake, client/patient attendance, and morbidity were frequently reported [47–49, 54, 65, 68, 69, 85, 92, 93, 99, 102]. Evaluations of feasibility, usability, and user acceptance were common in studies designing e-health products [42, 45, 57, 135–137]. Pre-existing validated questionnaires or instruments to evaluate outcomes were used in eight studies [44, 50, 65, 72, 139–141]. These evaluated health literacy [44], disease severity [65], disease-related stress [140, 142], quality of life [59], life satisfaction [59], self-efficacy [72, 140] perceptions of care and support [139, 140, 143], quality of mobile health apps [137], goals of care [142], and various other cognitive, emotional, behavioural, social or attitudinal elements of service providers and/or users [59, 72, 139, 140]. These were usually self-report instruments used as pre- and post-intervention comparisons with statistical analysis. A number of other authors developed their own questionnaires for pre-post comparisons or post-implementation evaluations.

#### Timing of evaluation

There was a paucity of longitudinal designs to determine what outcomes are achieved and sustained over the longer term [5]. Only a small number of authors evaluated outcomes over a longer period, for example, at 1 year [65], 2 years [41, 144], 3 years [69], and up to 6 years later [145]. It was sometimes difficult to determine at what point evaluation occurred, with authors typically reporting the length of the project overall but not necessarily when evaluations occurred.

#### Impact of public involvement on outcomes

Only one study was found which evaluated or measured the influence of public involvement on outcomes. That is, how the new product or service designs were different due to public involvement. Valaitis et al. [110] compared the ideas generated by public participants versus researchers and found nearly half (44.8%) of the ideas generated were novel, feasible, and could be integrated in some way. Some authors made comment on the value of public involvement, but these claims were not supported by objective evaluation. For example, Ruland et al. ([123], p.628-9) developed a software program to help children with cancer to communicate their symptoms, and noted the children involved in the design “…had many excellent and creative suggestions that the design team would not have thought of alone”. Similar observations were reported in three other studies [100, 107, 115].

#### Economic evaluations

Cost-benefit analyses were not reported in any studies, and economic evaluations were scarce. Locock et al. [107] evaluated the full costs of conducting an accelerated version of an experienced based co-design project compared to the standard method. In four studies, authors reported cost savings as a result of introducing a new model of care [41, 59, 85]. Airoldi [41] reported a 15% reduction in service costs. Forchuk et al. [59] reported an annual cost saving of $500,000 CAD for a new model of care. Cook et al. [139] determined a new model was budget neutral compared to a previous model but resulted in better outcomes and satisfaction for clients.

### Quality assessment

The MMAT quality assessment is shown in Additional file 3: Table S1. Only seven articles satisfied the first screening question: “Are there clear research questions?” ([146], p.2). Six were qualitative [84, 90, 100, 107, 111, 125] and generally demonstrated appropriateness of qualitative approach, data collection, analysis and interpretation. One was a non-randomized study [47], and demonstrated appropriate representation of the target population, measurements, and outcome data, accounted for confounders, and administered the intervention as intended.

The application of the critical appraisal guidelines for assessing the quality and impact of user involvement in health research [33] are shown in Additional file 3: Table S2. In many cases, there was insufficient detail reported to determine whether the criteria were met. The authors of the articles generally provided adequate detail about the rationale for, and level of, user involvement, and descriptions indicated ‘added-value’ of user involvement. However, these descriptions were usually reflective in nature or based on anecdotal stakeholder feedback rather than pre-planned evaluation or measurement. The articles were of lower quality for other criteria, indicating a lack of detail about recruitment strategies, user training, and methodological and ethical consideration. There was also limited detail regarding attempts to evaluate the user involvement component or involve users in the dissemination of findings.

## Discussion

This is the first systematic review to identify and synthesise what is known about health service outcomes of public involvement and how these outcomes were evaluated. The review addresses a major gap in current knowledge and reinforces the critical need to build a broader evidence base on what public involvement achieves and how best to enact it [10]. Interest in public involvement in health service design is rapidly increasing. In this review, over 60% of included studies were published in the last 5 years. Public involvement in health service is seen by some as a democratic and ethical right, with evaluation of outcomes, not a priority [147]. However, public involvement in health service design is resource intensive [12], and participants expect outcomes from their contributions [14, 16].

Robust evaluation is critical to build an evidence base that informs whether and how public involvement practices are linked to health service outcomes. Without robust evaluation, there is a risk that public involvement is simply a box-ticking exercise to comply with policy and accreditation standards [17, 18]. Evaluation informs what involvement methods are most effective [148] and encourages organisations to focus on the goals for public involvement and whether these have been achieved [149].

Despite the need for robust evaluation, a major finding of this review is that evaluating health service outcomes of public involvement is challenging [10, 150]. Authors commonly reported outputs (what results immediately from the activity) rather than outcomes (the result of the change) [151]. The majority of authors reported ‘added-value’ of public involvement (as per criterion 8 of the critical appraisal guidelines by Wright et al. [33]) and wide-ranging outcomes were clustered into categories of discrete products, improvement to health services, and system or policy level change/s. However, outcomes were evaluated in less than half of all studies.

There is a need for researchers to ensure that well designed, and robust evaluations are included in study designs a priori [152]. However, it is acknowledged that this can be challenging when impacts and outcomes cannot always be predetermined to guide appropriate evaluation methods. We encourage researchers to design broad evaluations and to be flexible enough to incorporate different methods and approaches in their design as their study develops. Russell, Fudge and Greenhalgh [153] reinforce the need for broad evaluation methods as narrow evaluation often fails to capture potential negative impacts and other more complex and longer-term outcomes, such as the impact on health service culture.

Evaluation should be linked to the study design and rationale for public involvement. For example, it is argued that public involvement can improve patient experience or satisfaction with services, and that potentially even “small-scale” changes can make a significant difference for patients [100]. Pre- and post- measures of these variables could be more frequently utilised in future evaluation studies to determine whether this is the case.

Given a rationale for public involvement in health service design is often accountability of resources [3, 14], or more efficient services [154, 155], it was surprising that few authors evaluated cost savings, and none conducted cost-benefit analyses.

In this review, there was a paucity of longitudinal studies that reported the sustainability of outcomes over time. Our findings aligned closely with what Russell, Fudge and Greenhalgh [153] identified as a major risk. That is, a propensity to focus on reporting outcomes that are more easily measured, short term and positive.

Staley [13] argues that public involvement is context-dependent and that evaluating outcomes of specific projects provides limited insight into broader questions about when and how involvement makes a difference or is worth doing. In this review, the majority of studies were conducted in single study sites, and there was a lack of consistency in how outcomes were evaluated. Although there does not yet appear to be a gold standard tool to measure health service outcomes from public involvement [20], tools do exist and have increased in number in the last decade [9]. It is not clear why these tools are not being widely utilised. Further work is needed to understand why this is the case, and to develop, test, and improve evaluation tools and measures. Awareness of these tools may be aided by such resources as the online toolkit produced by the Canadian organisation, Centre of Excellence on Partnership with Patients and the Public (CEPPP) [156].

We do note that there are studies currently underway, that appear to be well designed. The study protocol published by Kjellström et al. [157] may provide more evidence of health service outcomes and evaluation. The aim of their longitudinal, mixed-methods research is “…to explore, enhance and measure the value of co-production for improving the health and well-being of citizens” ([157], p.3), and one objective is to develop and test measures of co-production processes and outcomes. There is potential for national and transnational evaluations across services using consistent evaluation methods, and these could include randomised controlled trials. Large, cross country collaborations could result in the refinement or development of international tools for high quality transnational studies.

The characteristics of studies included in this review suggest a need for published evidence across a broader range of settings. Over half of the studies in this review were from the United Kingdom (UK). This may reflect UK history as one of the first countries to establish a welfare state following the second world war [158], and later, the influence of large government funding into public involvement programmes (e.g. INVOLVE) as part of neoliberal agendas. Major UK health research funding bodies require public involvement with a resultant flow on to academic publications [159]. More Australian studies might have been expected, given public involvement in the design of healthcare is mandated for health services via the National Safety and Quality Health Services Standards [155]. However, while the standards require evidence of consumer involvement for health service accreditation, there is no requirement for services to publish their activity. Additionally, the emphasis on public involvement in nationally competitive funding calls is only relatively recent.

Mental health service types made up over a quarter of the studies. This is not unexpected due to the long history of mental health service users driving health service reform, beginning with the psychiatric survivors’ movement in the 1970s [91]. Consumer involvement in all aspects of mental health service design and delivery has been enshrined in Australia’s National Mental Health Plans since 1992 [160]. There is clearly a need to broaden study context and focus.

There must be a greater focus on quality and reporting in studies of public involvement. Few authors in this review provided adequate details for rigorous assessment of quality appraisal. Authors should report greater detail about the definitions and conceptual underpinnings of public involvement and their study methods. Terminology has both conceptual and pragmatic implications and is necessary for the reader to understand the background and purpose of the study [161]. Without clarity of who is being involved, why, and how, it is hard to see the link between the public involvement element of the activity, and any outcome (expected or unexpected) that is achieved [162]. Reporting guidelines for public involvement do exist and should be utilised. For example, Staniszewska et al. [163] developed a reporting checklist, the Guidance for Reporting Involvement of Patients and the Public (GRIPP, and later updated as GRIPP2).

This review highlights that health service outcomes are sometimes published separately to the public involvement activity. Donetto, Tsianakas and Robert [12] found the majority of experience-based co-design projects included outcome evaluations, but these were often shared within the health organisation or at conferences rather than in academic peer-reviewed publications. In order to build a solid evidence base in the field the findings of future studies must be disseminated in a way that is readily available, such as publishing in open access journals, publishing outcome evaluations with public involvement activity descriptions, and via regular updates of systematic reviews.

### Limitations

This was the first review of its type to explore health service outcomes from public involvement and how these outcomes have been evaluated. It was important to capture all study types across a large body of literature on public involvement from multiple disciplines spanning decades. We consulted with a university librarian for advice on the search strategy and completed test searches of the primary databases in order to maximise the number of appropriate studies located. However, the different terminology and methodology in public involvement research has resulted in a large number of titles to screen and studies to review. Thorough cross-checking at all steps by a second reviewer was used to mitigate the potential for human error that is possible with such a large review. Future reviews could consider tighter refinement of terms as the quality of literature allows.

There is a level of subjectivity in quality appraisal tools and in the assignment of a level of public participation as per the IAP2. Bias was reduced by using a second reviewer and by resolving disagreements with consensus.

Finally, the continuous cycle of research and publication will inevitably have affected the results of this review. This review has shown a significant increase in studies in the last 5 years, and this growth is expected to continue. As an evolving field of research, regular updates of systematic reviews of evidence should be considered for new and relevant findings.

## Conclusion

Public involvement in health service design requires considerable investment of resources, including time and energy from the public. It is essential that empirical evidence demonstrates how public involvement improves health services and represents a worthwhile use of resources. While this systematic review demonstrates a range of health service outcomes arising from public involvement in health services design, these are predominantly known through descriptive reporting of immediate or shorter-term outcomes. The studies reviewed lacked consistency and varied considerably in quality. Formal evaluation of longer-term outcomes with objective measures is lacking, and there is an absence of large high quality transnational studies. Further research is required to understand what issues and challenges researchers face in evaluating impacts and how this could be improved. There is a critical need to improve the design and quality of studies. Longitudinal study designs which consider economic analyses are required. There must be ongoing development and use of valid and appropriate evaluation tools and reporting guidelines.

## Supplementary Information

**Additional file 1.** Search strategy example: MEDLINE database.

**Additional file 2.** Characteristics of included studies, the outcomes and impacts reported, and how they were evaluated.

**Additional file 3:** Quality apprisals of included studies.

## Data Availability

Articles included in the analysis are cited in the reference list.

## Abbreviations

CAD: Canadian dollar
CEPPP: Centre of Excellence on Partnership with Patients and the Public
GRIPP/GRIPP2: Guidance for Reporting Involvement of Patients and the Public
IAP2: International Association for Public Participation
MMAT: Mixed Methods Appraisal Tool
NHS: National Health Service (UK)
NICU: Neonatal Intensive Care Unit
OECD: Organisation for Economic Co-operation and Development
UK: United Kingdom
