# Supplementary material for: Evaluating health service outcomes of public involvement in health service design in high-income countries: a systematic review
Source: BMC Health Serv Res. 2021 Apr 20;21:364. doi: 10.1186/s12913-021-06319-1 (PMC8056601; doi:10.1186/s12913-021-06319-1)
Supplement: Supplementary file 1 — Additional file 1. Search strategy example: MEDLINE database. [file 12913_2021_6319_MOESM1_ESM.docx]

**Additional file 1**

**Search strategy example: Ovid MEDLINE database**

Note: "/" denotes a MeSH term, and "mp" a keyword).

1. health services (/)

2. health services (mp)

3. "health centre" or "health center" (mp)

4. community health (mp)

5. primary health care (/)

6. primary adj2 care (mp)

7. public health (mp)

8. Public health/

9. delivery of health care (/)

10.. health author$ (mp)

11. hospital$ (/)

12. "healthcare" or "health care" (mp)

13. 1 OR 2 OR 3 OR 4 OR 5 OR 6 OR 7 OR 8 OR 9 OR 10 OR 11 OR 12

14. involve$ (mp)

15. participat$ (mp)

16. collaborat$ (mp)

17. "co design" OR "codesign" (mp)

18. "co produc$" OR "coproduc$" (mp)

19. partner$ (mp)

20. engage$ (mp)

21. 14 OR 15 OR 16 OR 17 OR 18 OR 19 OR 20

22. community (mp)

23. patient (mp)

24. public (mp)

25. user (mp)

26. "service user" (mp)

27. citizen (mp)

28. "lay person" (mp)

29. consumer (mp)

30. client (mp)

31. 22 OR 23 OR 24 OR 25 OR 26 OR 27 OR 28 OR 29 OR 30

32. (health adj3 design) (mp) (untick map to SH and use brackets)

33. (service adj3 plan$) (mp)

34. (service adj3 design) (mp)

35. (priority adj3 setting) (mp)

36. 32 OR 33 OR 34 OR 35

37. 13 AND 21 AND 31 AND 36
