# Supplementary material for: Evaluating health service outcomes of public involvement in health service design in high-income countries: a systematic review
Source: BMC Health Serv Res. 2021 Apr 20;21:364. doi: 10.1186/s12913-021-06319-1 (PMC8056601; doi:10.1186/s12913-021-06319-1)
Supplement: Supplementary file 2 — Additional file 2. Characteristics of included studies, the outcomes and impacts reported, and how they were evaluated. [file 12913_2021_6319_MOESM2_ESM.docx]

**Additional file 2**

**Characteristics of included studies, the outcomes and impacts reported, and how they were evaluated.**

| **Study (primary author, year; country)** | **Study design** | **IAP2 (highest level)** | **Service setting and type** | **Health service outcomes** | **Type of health service outcome** | **Level of outcome (Unit= unit, team or service)** | **Status** | **If applicable, how were outcomes/impacts evaluated?** |
| --- | --- | --- | --- | --- | --- | --- | --- | --- |
| Adamou 2016 [1]; UK | Qual | Involve | Community, Mental health | Developed a framework to monitor client outcomes and guide service provision for adults with ADHD. | Product | Across organisations | Piloted |  |
| Airoldi 2013 [2];  UK | Qual | Consult | Multiple, Mental health | A strategy to reconfigure eating disorder services, which reduced spending on the program by >15%. | System | Across services | Evaluated | Reports a reduction in eating disorder program spending 2 years after implementation. No further detail provided. |
| Baggott 2015 [3];  USA | MM | Consult | Outpatients, Paediatrics | Testing of an app for children with cancer to communicate symptoms in a different country. | Product | Across organisations | Evaluated | Usability via time stamps and child and parent feedback (surveys and interviews). |
| Bauer 2018 [4]; USA | MM | Consult | Primary care, Mental health | Developed and deployed a mHealth app supporting collaborative care for rural patients with complex psychiatric conditions. Many changes to app design and functionality were made due to patient feedback. | Product | Across organisations | Implemented |  |
| Beauchamp 2017 [5];  Australia | MM | Involve | Community, Chronic/complex needs | Community volunteers trained in mentorship roles, e.g. in falls prevention program and as navigator; increased health literacy. | Service | Across organisations | Evaluated | Health literacy (Health Literacy Questionnaire) |
| Blanco 2019 [6]; Spain | MM | Consult | Multiple, Neurology | Developed, implemented and evaluated a micro Health Social Network (online paradigm) to assist in the therapeutic care of people with Parkinson's diseae and stroke, which is reported to be acceptable and efficient, and improves quality of care and peer support. | Product | Unit | Evaluated | Data logs of paradigm use, surveys, focus groups and interviews with users. Evaluated the following: user acceptance; productivity improvement; quality of service enhancement; fostering of social relations. |
| Borosund 2018 [7]; Norway | Qual | Consult | Outpatients, Oncology | Developed a prototype for a stress management app for use by cancer survivors. | Product | Tea, | Proposed |  |
| Boyd 2012 [8];  New Zealand | MM | Involve | Multiple, Oncology | Service improvements (e.g. information leaflets, communication guides, a patient held record, new patient gown); co-design website and toolkit. | Service | Unit | Implemented |  |
| Burbach 2019 [9]; UK | Qual | Involve | Community, Mental health | Developed service improvement plans with achievable and time-specific goals, and reported boosting staff morale. | Service | Unit | Proposed |  |
| Calvillo-Arbizu 2019 [10]; Spain | Qual | Consult | Community, Renal | Development of e-Nefro, an e-Health system available via app, web or telephone for the monitoring of renal patients at home. | Product | Unit | Piloted |  |
| Castensoe-Seidenfaden 2017 [11];  Denmark | MM | Collaborate | Outpatients, Endocrinology | Developed an app to support self-management of diabetes, which did not reduce glycated haemoglobin, or result in significant differences in self-reported self-management, hypoglycaemia, or hospitalizations [12]; but enhanced peer support, reduced feelings of loneliness, increased knowledge and skills in disease management, and was motivating for self-management [13]. | Product | Across organisations | Evaluated* | Subsequent RCT [12]: glycated haemoglobin; hypoglycaemia and hospitalizations; perceived competence in self-management (Perceived Competence in Diabetes Scale); perception of care provider to be autonomy-supportive (Health Care Climate Questionnaire); and perceived burden of diabetes-related problems (Problem Areas In Diabetes care survey) (baseline, 2, 7 and 12 months). Subsequent qualitative study [13]: interviews with young people and parents. |
| Chapman 2018 [14]; UK | Quant | Consult | Community, Chronic/complex needs | A community program for people with long term conditions, which significantly reduced number of emergency department visits and unplanned emergency admissions, with no significant change in planned elective admissions. | Service | Unit | Evaluated | Program cost; number of emergency department visits, unplanned emergency admissions, and planned elective admissions. |
| Chappel 2001 [15];  UK | MM | Consult | Multiple, Neurology | Developed a prioritisation process, the priorities and action plan, for stroke services. | System | Across organisations | Proposed |  |
| Cheng 2011 [16];  USA | MM | Involve | Community, Family planning | Expanded service offering at family planning clinic (e.g. routine screening, vaccination, laboratory tests, counselling, subsidized Weight Watchers) and improved process for referring patients externally. Patient volume increased. | Service | Across organisations | Evaluated | Service use; lab tests; BMI; health knowledge; patient satisfaction and staff perceptions (via survey). |
| Coad 2008 [17];  UK | Qual | Consult | Multiple, Paediatrics | Formation of a Youth Council, which contributed to service improvements and research designs. | Service | Unit | Implemented |  |
| Collins 2017 [18];  UK | MM | Involve | Community, Mental health | Formation of a Youth Council, re-design of child and adolescent mental health services to improve access, including development of a new service for 0-25y.o. Includes young people involved in staff recruitment. | System | Across services | Implemented |  |
| Cook 2007 [19];  USA | MM | Consult | Community, Mental health | 4 new service packages of psychosocial rehabilitation, a psychiatric emergency service, and a new assessment process. Resulted in a greater focus on recovery, increased peer support services, and reduced hospital readmissions. | System | Unit | Evaluated | Service use (community health programs, hospital readmissions); service access; consumer satisfaction. |
| Cook 2010 [20];  USA | Quant | Involve | Community, Mental health | Introduction of a pilot self-directed model of care where individual directs their funding allocation. Subsequent RCT found it resulted in higher user satisfaction with services and improved client outcomes, for no extra cost. | System | Across organisations | Evaluated* | Subsequent RCT [21] evaluated at baseline, 1yr, 2 yrs: client satisfaction with services (Client Satisfaction Questionnaire); self-perceived recovery (Recovery Assessment Scale); psychosocial status (Empowerment Scale); sense of personal control (Coping Mastery Scale);sense of autonomy-supported environments (Perceived Autonomy Support Scale); psychiatric and somatic symptoms (Brief Symptom Inventory's Global Severity Index); service utilisation; cost data. Compared outcomes statistically to baseline data. |
| Cooke 2014 [22];  UK | MM | Involve | Multiple, Respiratory | Consensus process of service user and professional views on service improvement for people with COPD. | Service | Across organisations | Proposed |  |
| Cooper 2016 [23];UK | Qual | Involve | Community, Mental health | Developed prioritised SMART goals for service improvement in community mental health. | Service | Unit | Proposed |  |
| Cotterell 2004 [24];  UK | Qual | Involve | Multiple, Oncology | Cancer Partnership Project resulted in users acting as a reference group; service improvements (e.g. information and education materials, standards for breaking bad news); redesigning a centre, advice for clinical trials. | Service | Across organisations | Implemented |  |
| Coylewright 2012 [25];  USA | Qual | Consult | Outpatients, Cardiology | Developed an individualised, patient-centred decision aid for adults with CAD, which resulted in increased patient knowledge and how informed patients felt about their choices, but did not significantly change extent to which clinicians engaged patients in decision making, or decisional quality [26]. | Product | Unit | Evaluated* | Evaluated in a subsequent randomized trial [26] the statistical differences between groups for: patient knowledge (questionnaire) and decisional conflict (Decisional Conflict Scale); shared decision making (Observing Patient Involvement in Decision Making (OPTION) scale). |
| Cramp 2006 [27];  UK | Qual | Consult | Multiple, Endocrinology | Local agreement reached for areas of service improvement, with 17 solutions and a proposed re-configuration of diabetes services. | System | Across organisations | Proposed |  |
| Crowley 2002 [28];  UK | MM | Collaborate | Primary care, All | Established a Community Action on Health Committee which developed new services, including funding (e.g. counselling service for ethnic minorities, community family support project, mobile youth services); increased service access (e.g. facilities for deaf community); improved dialogue between providers and users. | System | Across services | Evaluated | Questionnaires and interviews with local residents and community workers of their views and experiences, triangulated with observation, registers, documentary sources and diary monitoring exercises. |
| Csipke 2016 [29];  UK | MM | Involve | Inpatients – acute, Mental health | Developed and tested a tool to evaluate psychiatric ward environments, which identified service user and staff views and concerns. | Product | Unit | Piloted |  |
| Cushen 2004 [30];  UK | Qual | Collaborate | Multiple, Oncology | Generated solutions and action plan to improve cancer services (e.g. patient buddy system, improving clinic processes, breaking bad news guideline); new quality assurance model. | System | Across services | Partially implemented |  |
| Das 2013 [31];  Norway | Qual | Involve | Outpatients, Weight loss | Developed an e-health solution for adults undergoing weight loss programmes. | Product | Unit | Proposed |  |
| de Souza 2017 [32];  UK | Qual | Collaborate | Outpatients, Rheumatology | Formed an independent patient group which suggested service improvements (e.g. outpatient clinic experience which reduced waiting times, extended opening hours to phlebotomy, improved access to podiatry); started a new evening patient education programme; launched an app with hospital, clinic and patient information. | Service | Across services | Evaluated | Patient group and education evenings: mean attendance and attendee formal feedback; Service improvements: description of changes, mean clinic waiting times; App: download statistics and formal user feedback. |
| Dewar 2010 [33];  UK | Qual | Involve | Inpatients – acute, Multiple | Introduced touchpoint method to elicit stories from patients, resulting in service improvements (e.g. information booklet, methods to elicit regular feedback from patients and families). | Service | Unit | Evaluated | Staff and patient feedback of the method. |
| Diamond 2003 [34];  UK | MM | Collaborate | Multiple, Mental health | User involvement in service evaluation, and staff recruitment, training and induction; service improvements (e.g. meal choices, development of local outreach services, user-run café, introduced health checks with local GPs). | Service | Across services | Evaluated | Staff interviews and questionnaire audit against service user standards, to determine levels of user involvement in services and outcomes. |
| Dinniss 2007 [35];  UK | Quant | Collaborate | Multiple, Mental health | Developed an action plan for service development, resulting in service improvements (e.g. information packages, routine physical health screening check-up). | System | Across services | Implemented |  |
| Doherty 2018 [36]; UK | Qual | Consult | Outpatients, Women's health | Developed BrightSelf, a mobile app designed for use within a public health service for self-report of psychological wellbeing during pregnancy, which was positively received by women [37]. | Product | Unit | Evaluated* | Evaluated in a subsequent randomized control trial [37] feasibility of the app to engage women in mental health screening, with app usage data and participant surveys. |
| Dorrington 2015 [38];  Australia | Quant | Involve | Primary care, Women's health | Service improvements (e.g. culturally appropriate promotional materials, new clinic with female GP, improved recall letter, information leaflet), which resulted in significant increase in pap smear rates. | Service | Unit | Evaluated | Measured service use, and pap smear rates with statistical analysis. |
| Douglas 2005 [39];  UK | MM | Consult | Inpatients – acute, All | Developed 2 x indicator sets (internal and external) to appraise a proposed built environment design, which resulted in suggestions for design improvements. | Product | Across services | Proposed |  |
| Doyle 2016 [40];  Australia | MM | Collaborate | Community, All | Health promotion and community development programs, producing e.g. health promotional materials; activities which addressed environmental and social determinants of health, physical activity, nutrition and weight loss; development of participant skills and capacity; and strengthened social and cultural connections. | Service | Across organisations | Evaluated | Evaluated with an ecological framework. |
| Durey 2016 [41];  Australia | Qual | Collaborate | Multiple, All | Established Health Action Groups, resulting in new programs (e.g. diabetes education and podiatry care; antenatal care which significantly improved neonatal outcomes); increased employment opportunities for local Aboriginal people; strengthened relationships between community and health services; service improvements (e.g. more culturally appropriate, increased access, increased trust in services). | System | Across organisations | Evaluated | Stakeholder interview; Health outcomes (e.g. neonatal outcomes - not quantified). |
| Edwards 2016 [42];  UK | Qual | Collaborate | Inpatients – acute, Paediatrics | Proposed a framework for evaluating young adult and adolescent services. | Product | Across services | Proposed |  |
| Ennis 2014 [43]; UK | MM | Collaborate | Multiple, Mental health | Developed 'myhealthlocker', an electronic personal health record which includes care plans, health information, and Patient Reported Outcome Measures, and links primary and secondary care services, and service users. | Product | Across services | Evaluated | Feasibility questionnaire (predominantly quantitative yes/no and likert), but some open-ended questions. Results reported as mean scores, and data analysed with Fisher's exact test, t-tests and Spearman's correlations. Differences for age and gender explored. |
| Farr 2019 [44]; UK | Qual | Involve | Community, Mental health | Developed and piloted an electronic care pathway tool, which staff reported facilitated co-production of recovery-oriented care and crisis plans with service users [44]. | Product | Unit | Evaluated | In-depth staff interviews, with thematic analysis of data using Normalisation Process Theory as a framework. |
| Fitzgerald 2011 [45];  UK | Qual | Consult | Inpatients – subacute, Mental health | Changes to proposal for a new service development (e.g. design and refurbishment, changed proposed medication dispensing system). | Service | Unit | Implemented |  |
| Forchuk 1998 [46];  Canada | Qual | Consult | Multiple, Mental health | Implemented a new transitional care program for clients discharging from inpatient care to home, resulting in improved QOL, cost savings, increased staff work satisfaction. | System | Unit | Evaluated* | [46]: Interviews and questionnaires with staff and clients; service use. Another publication [47] reports statistical analysis of QOL (MacDonald, Sibbald and Hoare Satisfaction with Life (SWL) questionnaire); economic evaluation (community versus hospitalisation costs). |
| Gardener 2019 [48]; UK | Qual | Involve | Primary care, Respiratory | Developed 'The Support Needs Approach for Patients (SNAP) tool' for people with COPD to identify and communicate their support needs to health professionals. | Product | Unit | Piloted* |  |
| Hahn-Goldberg 2015 Hahn-Goldberg 2015 [49] & Hahn-Goldberg 2016 [50];  Canada | MM | Involve | Inpatients – acute, All | Developed a patient-oriented discharge summary, which improved patient experience and understanding of discharge instructions, with no change to staff workload. | Service | Across organisations | Evaluated* | A subsequent study [51] reporting piloting the tool at 8 hospitals (each modified the tool for local settings) and evaluated via patient and staff surveys: patient and provider experience; patient's perceived understanding of discharge instructions; staff time. |
| Han 2018 [52]; South Korea | MM | Consult | Multiple, Chronic/complex needs | Developed a collaborative multidisciplinary team care model 'DrugTEAM', for hospital inpatients and outpatients requiring multiple medications due to chronic disease. | Service | Across services | Proposed |  |
| Hickman 2019 [53]; Australia | Qual | Consult | Inpatients – acute, Surgical | Recommendations for the design of a technology assisted diet and exercise program for patients following liver transplant. | Service | Unit | Proposed |  |
| Hobson 2018 [54];  UK | Qual | Consult | Outpatients, Neurology | Developed a new telehealth system for monitoring patients' condition. | System | Unit | Piloted |  |
| Holloway 2006 [55];  UK | MM | Consult | Outpatients, Neurology | Developed new Care Pathway tools (information pack, Problems/Needs form, clinic summary and service record sheet), which was feasible and improved active engagement of patients in their care. Not clear if results in better illness management. | Service | Unit | Evaluated | Compared at baseline and 12 months: use of services, disease severity (Hoehn and Yahr scale). Post-implementation interviews with patients, carers and staff. |
| Irving 2018 [56];  UK | MM | Collaborate | Ambulance, Emergency care | Developed a set of ambulance performance measures. | Product | Unit | Proposed |  |
| Isenberg 2018 [57]; USA | MM | Collaborate | Outpatients, Surgical | Developed an Advance Care Plan video, which was found to be safe and feasible to implement but did not significantly change patient-surgeon communication for patients undergoing major surgery for advanced cancer [58]. | Product | Unit | Evaluated* | A subsequent RCT [58] compared: surgeon-patient conversations for advance care planning content and patient centredness, Hospital Anxiety and Depression Scales, the Iowa Goals of Care Survey; Helpfulness of the Video survey, patient and surgeon satisfaction surveys, and whether a medical decision maker had been designated. |
| Jackson 2003 [59];  UK | Qual | Collaborate | Primary care, All | Pilot of young people's clinic sessions at medical practice; funding to develop an information guide, a new health information and advice project at local high school, development of a video to raise awareness of young people's issues. Developed young people's confidence. | Service | Across organisations | Implemented |  |
| Jessup 2018 [60];  Australia | MM | Collaborate | Inpatients – acute, All | Identified 15 strategies to improve health literacy needs, including: changes to ways of delivering health messages, communication between care providers & hospitals, staff training. | Service | Across services | Partially implemented |  |
| Jones 2008 [61];  UK | Qual | Collaborate | Multiple, Neurology | Developed action plans and workgroups to implement service priorities, including: information resources, programme of rehabilitation and social activities. | System | Across organisations | Implemented |  |
| Kennedy 2014 [62];  UK | Qual | Consult | Primary care, Renal | Produced cartoons for use in a self-management guidebook. | Product | Unit | Piloted |  |
| Kenyon 2016 [63];  UK | Qual | Collaborate | Inpatients – acute, Women's health | Changes to care pathway, developed a new information leaflet, amendments to other patient information. | Service | Unit | Partially implemented |  |
| Kidd 2015 [64];  Australia | Qual | Collaborate | Multiple, Mental health | Developed workshops at the health service encouraging reflection on ideas of recovery, power imbalance and structural discrimination; recommendations made to service executive regarding consumer participation activities; and an action plan for service development. | Service | Unit | Proposed |  |
| Kilander 2019 [65]; Sweden | MM | Consult | Outpatients, Women's health | Introduced multiple service improvements to enhance contraceptive counselling and services at the time of an abortion. Improvements included: improved communication strategies, new and longer appointments, staff training. | Service | Across organisations | Evaluated | The proportion of women who chose an effective contraceptive method; the proportion of women commencing a LARC within 30 days post-abortion; health care professional's experience of the project. |
| Kildea 2018 [66];  Australia | MM | Consult | Outpatients, Women's health | Introduced a new program that is more accessible and culturally responsive, resulting in improved attendance rates and health outcomes compared to national proportions (lower pre-term births, C-sections, and NICU admissions). | Service | Unit | Evaluated | Attendance rates (service use, presentations in 1st trimester and having >5 visits antenatally) and health outcomes (pre-term births, C sections, NICU admissions, smoking cessation, breastfeeding rates) compared to Aboriginal and Torres Strait Islander figures nationally. Partially quantifies these outcomes. |
| Knight 2007 [67];  UK | MM | Consult | Outpatients, Oncology | Redesigned the care pathway to a 'one-stop clinic' model with target measures for performance. | Service | Unit | Implemented |  |
| Kohler 2017 [68];  Canada | Qual | Collaborate | Primary care, Endocrinology | Established infrastructure for patient and family engagement, including a definition, resource guide, team engagement and evaluation tools. | System | Across organisations | Piloted |  |
| Krist 2011 [69];  USA | MM | Consult | Primary care, Multiple | Developed an interactive patient health record, which was accepted by practices and patients, and helped patient manage their needs; subsequently resulted in higher rates of patients up to date with all indicated preventative screening tests, compared to nonusers [70]. | Service | Across organisations | Evaluated* | Percentage of patients using the health record (via record's database); health record usage patterns (Google Analytics); patient feedback (collected in the health record system). Subsequent randomized trial [70] evaluated: utlization and statistics of whether patients up to date with services (record's database and postal survey). |
| Larkin 2015 [71];  UK | Qual | Collaborate | Inpatients – acute, Mental health | Developed a service improvement action plan and priorities (e.g. patient journey action plan, ways to improve patient experience, model of recovery, recreational and activity programs, improved signage and access to ward spaces). | System | Unit | Partially implemented |  |
| Latif 2017 [72] & Manning 2017 [73];  UK | Qual | Involve | Inpatients – acute, Mental health | Produced e-learning training for nurses, which subsequently [73] improved staff knowledge, confidence and attitudes. | Product | Unit | Evaluated** | Evaluated in a subsequent study/publication [73]; pre and post-intervention evaluation of: attitudes towards self-harm (questionnaire); knowledge towards self-harm (questionnaire); confidence (Likert statements); self-efficacy (adapted version of the Self-efficacy Towards Helping Scale); clinical behaviour intention (Continuing Professional Development Reaction); qualitative interviews post-intervention. |
| Lo 2018 [74]; Australia | MM | Consult | Outpatients, Chronic/complex needs | Developed and implemented a new model of care for people with comorbid diabetes and chronic kidney disease, which subsequently improved integration of care and improved health and management of health [75]. | Service | Across organisations | Evaluated* | Evaluated in a subsequent study: experiences of patients and health care providers, strengths of model of care and areas for improvement (focus groups and semi-structured interviews) [75]. |
| Locock 2014 [76] & Boaz 2016 [77];  UK | MM | Collaborate | Inpatients – acute, Multiple | 48 service improvements (e.g. patient support groups, information leaflets, processes to enhance patient experience); changes to staff attitudes and organisational culture; determined the cost of the EBCD process used. Authors note suggestions would not have been considered without patient's input. | Service | Across organisations | Evaluated** | Ethnographic process evaluation; observations; interviews with staff; group interviews with patients; cost analysis; service improvement logs; participant evaluation questionnaires [76, 77]. |
| Lopatina 2019 [78]; Canada | Qual | Collaborate | Multiple, Rheumatology | Proposed strategies for a centralized intake for rheumatology care, and developed an evaluation framework including key performance indicators to measure quality of care and a patient experience survey. | System | Across services | Proposed |  |
| Lyles 2016 [79];  USA | Qual | Consult | Primary care, Chronic/complex needs | Produced tablet tool that helps patients plan their primary care visit. | Product | Across organisations | Proposed |  |
| Marshall 2006 [80];  UK | Qual | Involve | Primary care, All | Produced prototype guides to General Practice (paper and electronic versions). | Product | Across organisations | Proposed |  |
| McClelland 2018 [81]; UK | Qual | Involve | Community, Mental health | Developed a prototype mobile app for service users of an early intervention in psychosis service. | Product | Unit | Proposed |  |
| McWilliams 2018 [82]; USA | Qual | Involve | Multiple, Paediatrics | Developed 'Carolinas Asthma Coach', an interactive digital product to promote shared decision making, encourage self-management, and enable standardized evidence-based care. | Product | Across services | Piloted |  |
| Meldrum 2006 [83];  UK | Qual | Involve | Primary care, Sexual health | Developed a health service improvement strategy, which is reported to have increased service uptake. | System | Unit | Implemented |  |
| Melnick 2017 [84];  USA | Qual | Consult | Emergency, Neurology | Developed an app which integrates clinical decision support with a patient decision aid for ED patients with head injuries. | Product | Unit | Piloted |  |
| Olding 2018 [85]; Canada | Qual | Consult | Community, Drug and alcohol services | Developed a patient-oriented experience questionnaire to evaluate the redesign and reorientation of health service delivery. | Product | Across services | Proposed | Descriptive commentary: Authors reported people with diabetes "highlighted a number of locally important issues that should be incorporated into the questionnaire to enhance the relevance and quality of data collection." p.20. |
| Outlaw 2018 [86]; UK | Qual | Involve | Outpatients, Chronic/complex needs | Identified priorities and proposed solutions for service improvements, including for communication processes, roles of staff, new information sheets, and physical environment. | Service | Unit | Proposed | Identified priorities and proposed solutions for service improvements, including for communication processes, roles of staff, new information sheets, and physical environment. |
| Owens 2011 [87];  UK | Qual | Involve | Emergency, Mental health | Designed a personalised text messaging system to support people who self-harm, which was subsequently implemented for feasibility evaluation [88], but had issues with participant recruitment and retention, possibly due to delivery setting. | System | Unit | Evaluated* | Subsequent study [88] used normalisation process theory to evaluate feasibility of the intervention, and whether worthwhile to undertake a full trial. Evaluated with field notes, and focus group and interviews with clinicians and managers. |
| Pilgrim 1998 [89];  UK | Qual | Involve | Community, Mental health | Established priorities for service improvements; implemented service changes (service information booklet, and appointment of an advocate); skill development of group members. | Service | Unit | Implemented |  |
| Piper 2012 [90];  Australia | Qual | Collaborate | Emergency, Emergency care | Service and process improvements (e.g. staffing, physical infrastructure, staff training, patient FAQ sheets, and processes). Spread of EBCD processes to other units in same facility. | System | Across organisations | Evaluated | Interviews with clinicians, managers, patients and high-level project managers; evaluations of project team reports, data and documents contextualising EBCD programmes. |
| Powell 1994 [91];  UK | Qual | Consult | Inpatients – acute, Geriatrics | Service improvements (e.g. new information leaflet, display of staff photographs, new programme empowering patients to be charge of their own care); designed criterion based survey to integrate user satisfaction with clinical audit. | Service | Unit | Implemented |  |
| Probst 2018 [92];  USA | Qual | Consult | Emergency, Cardiology | Developed a prototype of a paper-based decision aid for ED patients with syncope. | Product | Unit | Proposed |  |
| Reaume-Zimmer 2019 [93]; Canada | Qual | Collaborate | Multiple, Mental health | Service transformation of youth mental health services through establishment of a youth-friendly space, integration of services and community partnerships, and multiple strategies to improve awareness of and access to services. | Service | Across organisations | Implemented | Number of youth seeking mental healthcare. |
| Robinson 2019 [94]; UK | MM | Consult | Multiple, Trauma | Produced cluster maps to evaluate a major trauma service for musculoskeletal injuries, determine which issues matter to patients and identify areas for targeted service improvement, and could be used as a benchmark for tracking any improvements. | Service | Unit | Proposed |  |
| Romm 2019 [95]; Norway | Quant | Involve | Outpatients, Mental health | Implemented an Early Intervention Service for psychosis, with strategies to improve access, communication and awareness, and outreach services. | Service | Unit | Evaluated | Analysis of phone calls to the Early Intervention in Psychosis phone service |
| Ruland 2008 [96];  Norway | Qual | Involve | Multiple, Paediatrics | An app for children with cancer to communicate symptoms (Sisom). It was subsequently found to be feasible (with some refinements) for children in USA [97], Sweden [98], and Canada [99, 100]; symptoms were more likely to be reported and was preferred compared to another symptom reporting checklist [3], and was perceived as easy and useful [3, 100]. | Product | Across organisations | Evaluated*** | Authors note children had ideas they would not have come up with in developing the app. Evaluated in subsequent studies: usability with children in USA [97], Sweden [98] and Canada [99, 100], including language validation as required; comparison of symptom reporting of Sisom with a validated symptom checklist (Memorial Symptom Assessment Scale), and usability via time stamps and child and parent feedback (surveys and interviews) [3]. |
| Taylor 2015 [101];  UK | Qual | Consult | Community, Chronic/complex needs | Redesigned telehealth processes (e.g. referral and discharge processes, assessment and review of users, improved data sharing); service improvement (e.g. monitoring and triage of patients); awareness of service. | Service | Unit | Implemented |  |
| Thomson 2015 [102];  UK | Qual | Involve | Outpatients, Neurology | Prototyped and tested service improvements (e.g. clinic guide for new patients, clinic dictionary, walking map); volunteer service which has developed into a Patient Advisory Group. | Service | Unit | Implemented |  |
| Tsianakas 2012 [103];  UK | Qual | Collaborate | Multiple, Oncology | Implemented a range of service improvements to improve patient experience (e.g. physical environment, appointment processes, service opening hours, new information resources); changes to staff induction; sharing of data across sites; new group education programs and new nurse clinics. | Service | Across services | Evaluated | Description of outputs and outcomes as perceived by staff. |
| Tsimicalis 2018 [100];  Canada | MM | Consult | Multiple, Paediatrics | Testing of an app for children with cancer to communicate symptoms in a different country. | Product | Across organisations | Evaluated | Children's verbalisations and device signals (usability software) and feedback (interviews). |
| Valaitis 2019 [104]; Canada | Qual | Consult | Primary care, Geriatrics | Designed and implemented Health TAPESTRY: a primary health care intervention to promote optimal aging. | Service | Across organisations | Evaluated | Number/percentage and description of ideas generated that were 'novel' (had not been considered by the research team). |
| Warnestal 2017 [105];  Sweden | Qual | Involve | Community, Paediatrics | Development of a digital peer support service prototype with the creation of child personas. | Product | Unit | Piloted |  |
| Woods 2018 [106]; Australia | Qual | Consult | Outpatients, Cardiology | Developed a mobile application for self-management as an adjunct to specialist care for patients with heart failure, which patients viewed as potentially valuable for daily condition management and communication with health professionals [107]. | Product | Unit | Evaluated* | Woods et al. [107] evaluated patient's experience of the app (mixed methods usability study using a modified Mobile Application Rating Scale and semi-structured interviews); and Woods et al. [108] evaluated participant experiences of co-design (interviews with clinicians, patients and caregivers). |
| Yu 2019 [109]; Canada | Qual | Consult | Primary care, Endocrinology | Development of *MyDiabetesPlan,* a goal setting patient-decision aid toolkit to facilitate shared decision making. Developed an implementation strategy including identifying clinical champion, staff training session and training resources, and integration with the electronic medical record. | Product | Unit | Piloted |  |

**Abbreviations**: *Qual* Qualitative, *MM* Mixed Methods, *Quant* Quantitative, *Evaluated** Evaluated (exclusively or additionally) in a subsequent project/article not included in the review, *Evaluated*** Evaluated (exclusively or additionally) in a subsequent article included in the review, *Evaluated**** Evaluated in articles included and not included in the review, *UK* United Kingdom, *USA* United States of America, *app* Application, *RCT* Randomised Controlled Trial, *GP* General Practitioner, *QOL* Quality Of Life, *NICU* Neonatal Intensive Care Unit, *EBCD* Experienced Based Co-Design, *ED* Emergency Department, *FAQ* Frequently Asked Questions.

**References**

1. Adamou M, Graham K, MacKeith J, Burns S, Emerson L-M: **Advancing services for adult ADHD: the development of the ADHD Star as a framework for multidisciplinary interventions**. In: *BMC Health Serv Res.* vol. 16; 2016.

2. Airoldi M: **Disinvestments in Practice: Overcoming Resistance to Change through a Sociotechnical Approach with Local Stakeholders**. *J Health Polit Policy Law* 2013, **38**(6):1149-1171.

3. Baggott C, Baird J, Hinds P, Ruland CM, Miaskowski C: **Evaluation of Sisom: A computer-based animated tool to elicit symptoms and psychosocial concerns from children with cancer**. *Eur J Oncol Nurs* 2015, **19**(4):359-369.

4. Bauer AM, Hodsdon S, Bechtel JM, Fortney JC: **Applying the Principles for Digital Development: Case Study of a Smartphone App to Support Collaborative Care for Rural Patients With Posttraumatic Stress Disorder or Bipolar Disorder**. *J Med Internet Res* 2018, **20**(6):e10048.

5. Beauchamp A, Batterham RW, Dodson S, Astbury B, Elsworth GR, McPhee C, Jacobson J, Buchbinder R, Osborne RH: **Systematic development and implementation of interventions to OPtimise Health Literacy and Access (Ophelia)**. In: *BMC Public Health.* vol. 17; 2017.

6. Blanco T, Casas R, Marco A, Martinez I: **Micro ad-hoc Health Social Networks (uHSN). Design and evaluation of a social-based solution for patient support**. *J Biomed Inform* 2019, **89**:68-80.

7. Borosund E, Mirkovic J, Clark MM, Ehlers SL, Andrykowski MA, Bergland A, Westeng M, Solberg Nes L: **A Stress Management App Intervention for Cancer Survivors: Design, Development, and Usability Testing**. *JMIR Form Res* 2018, **2**(2):e19.

8. Boyd H, McKernon S, Mullin B, Old A: **Improving healthcare through the use of co-design**. *N Z Med J* 2012, **125**(1357):76-87.

9. Burbach FR, Amani SK: **Appreciative enquiry peer review improving quality of services**. *Int J Health Care Qual Assur* 2019, **32**(5):857-866.

10. Calvillo-Arbizu J, Roa-Romero LM, Estudillo-Valderrama MA, Salgueira-Lazo M, Areste-Fosalba N, del-Castillo-Rodriguez NL, Gonzalez-Cabrera F, Marrero-Robayna S, Lopez-de-la-Manzana V, Roman-Martinez I: **User-centred design for developing e-Health system for renal patients at home (AppNephro)**. *Int J Med Inform* 2019, **125**:47-54.

11. Castensøe-Seidenfaden P, Husted GR, Teilmann G, Hommel E, Olsen BS, Kensing F: **Designing a Self-Management App for Young People With Type 1 Diabetes: Methodological Challenges, Experiences, and Recommendations**. In: *JMIR Mhealth Uhealth.* vol. 5; 2017: e124.

12. Castensøe-Seidenfaden P, Husted GR, Jensen AK, Hommel E, Olsen B, Pedersen-Bjergaard U, Kensing F, Teilmann G: **Testing a smartphone app (Young with Diabetes) to improve self-management of diabetes over 12 months: randomized controlled trial**. In: *JMIR Mhealth Uhealth.* vol. 6; 2018: e141.

13. Husted GR, Weis J, Teilmann G, Castensøe-Seidenfaden P: **Exploring the influence of a smartphone app (young with diabetes) on young people’s self-management: qualitative study**. In: *JMIR Mhealth Uhealth.* vol. 6; 2018: e43.

14. Chapman H, Farndon L, Matthews R, Stephenson J: **Okay to Stay? A new plan to help people with long-term conditions remain in their own homes**. *Prim Health Care Res Dev* 2018, **20**.

15. Chappel D, Bailey J, Stacy R, Rodgers H, Thomson R: **Implementation and evaluation of local-level priority setting for stroke**. *Public Health* 2001, **115**(1):21-29.

16. Cheng D, Patel P: **Optimizing Women’s Health in a Title X Family Planning Program, Baltimore County, Maryland, 2001-2004**. *Prev Chronic Dis* 2011, **8**(6):A126.

17. Coad J, Flay J, Aspinall M, Bilverstone B, Coxhead E, Hones B: **Evaluating the impact of involving young people in developing children’s services in an acute hospital trust**. *J Clin Nurs* 2008, **17**(23):3115-3122.

18. Collins R, Notley C, Clarke T, Wilson J, Fowler D: **Participation in developing youth mental health services: “Cinderella service” to service re-design**. *J Public Ment Health* 2017, **16**(4):159-168.

19. Cook JA, Ruggiero K, Shore S, Daggett P, Butler SB: **Public-academic collaboration in the application of evidence-based practice in Texas mental health system redesign**. *Int J Ment Health* 2007, **36**(2):36-49.

20. Cook JA, Shore SE, Burke-Miller JK, Jonikas JA, Ferrara M, Colegrove S, Norris WT, Ruckdeschel B, Batteiger AP, Ohrtman M: **Participatory action research to establish self-directed care for mental health recovery in Texas**. *Psychiatr Rehabil J* 2010, **34**(2):137-144.

21. Cook JA, Shore S, Burke-Miller JK, Jonikas JA, Hamilton M, Ruckdeschel B, Norris W, Markowitz AF, Ferrara M, Bhaumik D: **Mental health self-directed care financing: efficacy in improving outcomes and controlling costs for adults with serious mental illness**. *Psychiatr Serv* 2019, **70**(3):191-201.

22. Cooke M, Campbell M: **Comparing patient and professional views of expected treatment outcomes for chronic obstructive pulmonary disease: A Delphi study identifies possibilities for change in service delivery in England, UK**. *J Clin Nurs* 2014, **23**(13-14):1990-2002.

23. Cooper K, Gillmore C, Hogg L: **Experience-based co-design in an adult psychological therapies service**. *J Ment Health* 2016, **25**(1):36-40.

24. Cotterell P, Sitzia J, Richardson A: **Evaluating partnerships with cancer patients**. *Practice Nursing* 2004, **15**(9):430-435.

25. Coylewright M, Shepel K, LeBlanc A, Pencille L, Hess E, Shah N, Montori VM, Ting HH: **Shared decision making in patients with stable coronary artery disease: PCI choice**. *PLoS One* 2012, **7**(11):e49827.

26. Coylewright WM, Dick DS, Zmolek PB, Askelin MJ, Hawkins HE, Branda HM, Inselman HJ, Zeballos-Palacios HC, Shah HN, Hess HE *et al*: **PCI choice decision aid for stable coronary artery disease: A randomized trial**. *Circ Cardiovasc Qual Outcomes* 2016, **9**(6):767-776.

27. Cramp G: **Development of an integrated and sustainable rural service for people with diabetes in the Scottish Highlands**. *Rural Remote Health* 2006, **6**(1):422.

28. Crowley P, Green J, Freake D, Drinkwater C: **Primary Care Trusts involving the community: is community development the way forward?** *J Manag Med* 2002, **16**(4):311-322.

29. Csipke E, Papoulias C, Vitoratou S, Williams P, Rose D, Wykes T: **Design in mind: eliciting service user and frontline staff perspectives on psychiatric ward design through participatory methods**. *J Ment Health* 2016, **25**(2):114-121.

30. Cushen N, South J, Kruppa S: **Patients as teachers: the patient's role in improving cancer services**. *Prof Nurse* 2004, **19**(7):395-399.

31. Das A, Svanæs D: **Human-centred methods in the design of an e-health solution for patients undergoing weight loss treatment**. *Int J Med Inform* 2013, **82**(11):1075-1091.

32. de Souza S, Galloway J, Simpson C, Chura R, Dobson J, Gullick NJ, Steer S, Lempp H: **Patient involvement in rheumatology outpatient service design and delivery: a case study**. *Health Expect* 2017, **20**(3):508-518.

33. Dewar B, Mackay R, Smith S, Pullin S, Tocher R: **Use of emotional touchpoints as a method of tapping into the experience of receiving compassionate care in a hospital setting**. *J Res Nurs* 2010, **15**(1):29-41.

34. Diamond B, Parkin G, Morris K, Bettinis J, Bettesworth C: **User involvement: substance or spin?** *J Ment Health* 2003, **12**(6):613-626.

35. Dinniss S, Roberts G, Hubbard C, Hounsell J, Webb R: **User-led assessment of a recovery service using DREEM**. *Psychiatric Bulletin* 2007, **31**(4):124-127.

36. Doherty K, Barry M, Marcano-Belisario J, Arnaud B, Morrison C, Car J, Doherty G: **A Mobile App for the Self-Report of Psychological Well-Being During Pregnancy (BrightSelf): Qualitative Design Study**. *JMIR Ment Health* 2018, **5**(4):e10007.

37. Doherty K, Marcano-Belisario J, Cohn M, Mastellos N, Morrison C, Car J, Doherty G: **Engagement with Mental Health Screening on Mobile Devices: Results from an Antenatal Feasibility Study**. In: *Proceedings of the 2019 CHI Conference on Human Factors in Computing Systems: 2019*; 2019: 1-15.

38. Dorrington MS, Herceg A, Douglas K, Tongs J, Bookallil M: **Increasing Pap smear rates at an urban Aboriginal Community Controlled Health Service through translational research and continuous quality improvement**. *Aust J Prim Health* 2015, **21**(4):417-422.

39. Douglas CH, Douglas MR: **Patient-centred improvements in health-care built environments: perspectives and design indicators**. *Health Expect* 2005, **8**(3):264-276.

40. Doyle J, Atkinson-Briggs S, Atkinson P, Firebrace B, Calleja J, Reilly R, Cargo M, Riley T, Crumpen T, Rowley K: **A prospective evaluation of first people’s health promotion program design in the goulburn-murray rivers region**. In: *BMC Health Serv Res.* vol. 16; 2016: 645.

41. Durey A, McEvoy S, Swift-Otero V, Taylor K, Katzenellenbogen J, Bessarab D: **Improving healthcare for Aboriginal Australians through effective engagement between community and health services**. In: *BMC Health Serv Res.* vol. 16; 2016.

42. Edwards M, Lawson C, Rahman S, Conley K, Phillips H, Uings R: **What does quality healthcare look like to adolescents and young adults? Ask the experts!** *Clin Med (Lond)* 2016, **16**(2):146-151.

43. Ennis L, Robotham D, Denis M, Pandit N, Newton D, Rose D, Wykes T: **Collaborative development of an electronic Personal Health Record for people with severe and enduring mental health problems**. *BMC Psychiatry* 2014, **14**(1):305.

44. Farr M, Pithara C, Sullivan S, Edwards H, Hall W, Gadd C, Walker J, Hebden N, Horwood J: **Pilot implementation of co-designed software for co-production in mental health care planning: a qualitative evaluation of staff perspectives**. *J Ment Health* 2019, **28**(5):495-504.

45. Fitzgerald MM, Kirk GD, Bristow CA: **Description and evaluation of a serious game intervention to engage low secure service users with serious mental illness in the design and refurbishment of their environment**. *J Psychiatr Ment Health Nurs* 2011, **18**(4):316-322.

46. Forchuk C, Schofield R, Martin M-L, Sircelj M, Woodcox V, Jewell J, Valledor T, Overby B, Chan L: **Bridging the discharge process: Staff and client experiences over time**. *J Am Psychiatr Nurses Assoc* 1998, **4**(4):128-133.

47. Forchuk C, Chan L, Schofield R, Martin M-L, Sircelj M, Woodcox V, Jewell J, Valledor T, Overby B: **Bridging the discharge process**. *Can Nurse* 1998, **94**(3):22-26.

48. Gardener A, Ewing G, Farquhar M: **Enabling patients with advanced chronic obstructive pulmonary disease to identify and express their support needs to health care professionals: A qualitative study to develop a tool**. *Palliat Med* 2019, **33**(6):663-675.

49. Hahn‐Goldberg S, Okrainec K, Huynh T, Zahr N, Abrams H: **Co‐creating patient‐oriented discharge instructions with patients, caregivers, and healthcare providers**. *J Hosp Med* 2015, **10**(12):804-807.

50. Hahn-Goldberg S, Damba C, Solomon F, Okrainec K, Abrams H, Huynh T: **Using co-design methods to create a patient-oriented discharge summary**. *J Clin Outcomes Manag* 2016, **23**(7):321-328.

51. Hahn-Goldberg S, Okrainec K, Damba C, Huynh T, Lau D, Maxwell J, McGuire R, Yang L, Abrams HB: **Implementing Patient-Oriented Discharge Summaries (PODS): a multisite pilot across early adopter hospitals**. *Healthcare Quarterly* 2016, **19**(1):42-48.

52. Han N, Han SH, Chu H, Kim J, Rhew KY, Yoon JH, Je NK, Rhie SJ, Ji E, Lee E *et al*: **Service design oriented multidisciplinary collaborative team care service model development for resolving drug related problems**. *PLoS One* 2018, **13**(9).

53. Hickman IJ, Coran D, Wallen MP, Kelly J, Barnett A, Gallegos D, Jarrett M, McCoy SM, Campbell KL, Macdonald GA: **'Back to Life'-Using knowledge exchange processes to enhance lifestyle interventions for liver transplant recipients: A qualitative study**. *Nutr Diet* 2019, **76**(4):399-406.

54. Hobson EV, Baird WO, Partridge R, Cooper CL, Mawson S, Quinn A, Shaw PJ, Walsh T, Wolstenholme D, McDermott CJ: **The TiM system: developing a novel telehealth service to improve access to specialist care in motor neurone disease using user-centered design**. *Amyotrophic Lateral Sclerosis and Frontotemporal Degeneration* 2018:1-11.

55. Holloway M: **Traversing the network: a user‐led Care Pathway approach to the management of Parkinson's disease in the community**. *Health Soc Care Community* 2006, **14**(1):63-73.

56. Irving A, Turner J, Marsh M, Broadway-Parkinson A, Fall D, Coster J, Siriwardena AN: **A coproduced patient and public event: An approach to developing and prioritizing ambulance performance measures**. *Health Expect* 2018, **21**(1):230-238.

57. Isenberg SR, Crossnohere NL, Patel MI, Conca-Cheng A, Bridges JFP, Swoboda SM, Smith TJ, Pawlik TM, Weiss M, Volandes AE *et al*: **An advance care plan decision support video before major surgery: a patient- and family-centred approach**. *BMJ Support Palliat Care* 2018, **8**(2):229‐236.

58. Aslakson RA, Isenberg SR, Crossnohere NL, Conca-Cheng AM, Moore M, Bhamidipati A, Mora S, Miller J, Singh S, Swoboda SM: **Integrating Advance Care Planning Videos into Surgical Oncologic Care: A Randomized Clinical Trial**. *J Palliat Med* 2019, **22**(7):764-772.

59. Jackson AM: **‘Follow the Fish’: involving young people in primary care in Midlothian**. *Health Expect* 2003, **6**(4):342-351.

60. Jessup RL, Osborne RH, Buchbinder R, Beauchamp A: **Using co-design to develop interventions to address health literacy needs in a hospitalised population**. In: *BMC Health Serv Res.* vol. 18; 2018.

61. Jones SP, Auton MF, Burton CR, Watkins CL: **Engaging service users in the development of stroke services: an action research study**. *J Clin Nurs* 2008, **17**(10):1270-1279.

62. Kennedy A, Rogers A, Blickem C, Daker-White G, Bowen R: **Developing cartoons for long-term condition self-management information**. In: *BMC Health Serv Res.* vol. 14; 2014.

63. Kenyon SL, Johns N, Duggal S, Hewston R, Gale N: **Improving the care pathway for women who request Caesarean section: an experience-based co-design study**. In: *BMC Pregnancy Childbirth.* vol. 16; 2016.

64. Kidd S, Kenny A, McKinstry C: **Exploring the meaning of recovery-oriented care: An action-research study**. *Int J Ment Health Nurs* 2015, **24**(1):38-48.

65. Kilander H, Brynhildsen J, Alehagen SW, Fagerkrantz A, Thor J: **Collaboratively seeking to improve contraceptive counselling at the time of an abortion: a case study of quality improvement efforts in Sweden**. *BMJ Sex Reprod Health* 2019, **45**(3):190-199.

66. Kildea S, Hickey S, Nelson C, Currie J, Carson A, Reynolds M, Wilson K, Kruske S, Passey M, Roe Y: **Birthing on Country (in Our Community): a case study of engaging stakeholders and developing a best-practice Indigenous maternity service in an urban setting**. *Aust Health Rev* 2018, **42**(2):230-238.

67. Knight JA: **Change management in cancer care: a one-stop gynaecology clinic**. *Br J Nurs* 2007, **16**(18):1122-1126.

68. Kohler G, Sampalli T, Ryer A, Porter J, Wood L, Bedford L, Higgins-Bowser I, Edwards L, Christian E, Dunn S: **Bringing value-based perspectives to care: including patient and family members in decision-making processes**. *Int J Health Policy Manag* 2017, **6**(11):661-668.

69. Krist AH, Peele E, Woolf SH, Rothemich SF, Loomis JF, Longo DR, Kuzel AJ: **Designing a patient-centered personal health record to promote preventive care**. In: *BMC Med Inform Decis Mak.* vol. 11; 2011.

70. Krist AH, Woolf SH, Rothemich SF, Johnson RE, Peele JE, Cunningham TD, Longo DR, Bello GA, Matzke GR: **Interactive preventive health record to enhance delivery of recommended care: a randomized trial**. *The Annals of Family Medicine* 2012, **10**(4):312-319.

71. Larkin M, Boden ZV, Newton E: **On the brink of genuinely collaborative care experience-based co-design in mental health**. *Qual Health Res* 2015, **25**(11):1463-1476.

72. Latif A, Carter T, Rychwalska-Brown L, Wharrad H, Manning J: **Co-producing a digital educational programme for registered children’s nurses to improve care of children and young people admitted with self-harm**. *J Child Health Care* 2017, **21**(2):191-200.

73. Manning JC, Carter T, Latif A, Horsley A, Cooper J, Armstrong M, Crew J, Wood D, Callaghan P, Wharrad H: **‘Our Care through Our Eyes’. Impact of a co-produced digital educational programme on nurses’ knowledge, confidence and attitudes in providing care for children and young people who have self-harmed: a mixed-methods study in the UK**. In: *BMJ Open.* vol. 7; 2017: e014750.

74. Lo C, Zimbudzi E, Teede H, Cass A, Fulcher G, Gallagher M, Kerr PG, Jan S, Johnson G, Mathew T *et al*: **Models of care for co‐morbid diabetes and chronic kidney disease**. *Nephrology* 2018, **23**(8):711-717.

75. Zimbudzi E, Lo C, Robinson T, Ranasinha S, Teede HJ, Usherwood T, Polkinghorne KR, Kerr PG, Fulcher G, Gallagher M: **The impact of an integrated diabetes and kidney service on patients, primary and specialist health professionals in Australia: A qualitative study**. *PLoS One* 2019, **14**(7).

76. Locock L, Robert G, Boaz A, Vougioukalou S, Shuldham C, Fielden J, Ziebland S, Gager M, Tollyfield R, Pearcey J: **Using a national archive of patient experience narratives to promote local patient-centered quality improvement: an ethnographic process evaluation of ‘accelerated’experience-based co-design**. *J Health Serv Res Policy* 2014, **19**(4):200-207.

77. Boaz A, Robert G, Locock L, Sturmey G, Gager M, Vougioukalou S, Ziebland S, Fielden J: **What patients do and their impact on implementation: An ethnographic study of participatory quality improvement projects in English acute hospitals**. *J Health Organ Manag* 2016, **30**(2):258-278.

78. Lopatina E, Miller JL, Teare SR, Marlett NJ, Patel J, Barber CEH, Mosher DP, Wasylak T, Woodhouse LJ, Marshall DA: **The voice of patients in system redesign: A case study of redesigning a centralized system for intake of referrals from primary care to rheumatologists for patients with suspected rheumatoid arthritis**. *Health Expect* 2019, **22**(3):348-363.

79. Lyles CR, Altschuler A, Chawla N, Kowalski C, McQuillan D, Bayliss E, Heisler M, Grant RW: **User-centered design of a tablet waiting room tool for complex patients to prioritize discussion topics for primary care visits**. In: *JMIR Mhealth Uhealth.* vol. 4; 2016: e108.

80. Marshall M, Noble J, Davies H, Waterman H, Walshe K, Sheaff R, Elwyn G: **Development of an information source for patients and the public about general practice services: an action research study**. *Health Expect* 2006, **9**(3):265-274.

81. McClelland GT, Fitzgerald M: **A participatory mobile application (app) development project with mental health service users and clinicians**. *Health Educ J* 2018, **77**(7):815-827.

82. McWilliams A, Reeves K, Shade L, Burton E, Tapp H, Courtlandt C, Gunter A, Dulin MF: **Patient and Family Engagement in the Design of a Mobile Health Solution for Pediatric Asthma: Development and Feasibility Study**. *JMIR Mhealth Uhealth* 2018, **6**(3):e68.

83. Meldrum J, Pringle A: **Sex, lives and videotape**. *The journal of the Royal Society for the Promotion of Health* 2006, **126**(4):172-177.

84. Melnick ER, Hess EP, Guo G, Breslin M, Lopez K, Pavlo AJ, Abujarad F, Powsner SM, Post LA: **Patient-centered decision support: formative usability evaluation of integrated clinical decision support with a patient decision aid for minor head injury in the emergency department**. *J Med Internet Res* 2017, **19**(5):1-12.

85. Olding M, Hayashi K, Pearce L, Bingham B, Buchholz M, Gregg D, Hamm D, Shaver L, McKendry R, Barrios R *et al*: **Developing a patient-reported experience questionnaire with and for people who use drugs: A community engagement process in Vancouver's Downtown Eastside**. *Int J Drug Policy* 2018, **59**:16-23.

86. Outlaw P, Tripathi S, Baldwin J: **Using patient experiences to develop services for chronic pain**. *Br J Pain* 2018, **12**(2):122-131.

87. Owens C, Farrand P, Darvill R, Emmens T, Hewis E, Aitken P: **Involving service users in intervention design: a participatory approach to developing a text-messaging intervention to reduce repetition of self-harm**. *Health Expect* 2011, **14**(3):285-295.

88. Owens C, Charles N: **Implementation of a text-messaging intervention for adolescents who self-harm (TeenTEXT): a feasibility study using normalisation process theory**. In: *Child Adolesc Psychiatry Ment Health.* vol. 10. London; 2016.

89. Pilgrim D, Waldron L: **User involvement in mental health service development: how far can it go?** *J Ment Health* 1998, **7**(1):95-104.

90. Piper D, Iedema R, Gray J, Verma R, Holmes L, Manning N: **Utilizing experience-based co-design to improve the experience of patients accessing emergency departments in New South Wales public hospitals: An evaluation study**. *Health Serv Manage Res* 2012, **25**(4):162-172.

91. Powell J, Lovelock R, Bray J, Philp I: **Involving consumers in assessing service quality: benefits of using a qualitative approach**. *Qual Health Care* 1994, **3**(4):199-202.

92. Probst MA, Hess EP, Breslin M, Frosch DL, Sun BC, Langan M-N, Richardson LD: **Development of a Patient Decision Aid for Syncope in the Emergency Department: the SynDA Tool**. *Acad Emerg Med* 2018, **25**(4):425-433.

93. Reaume-Zimmer P, Chandrasena R, Malla A, Joober R, Boksa P, Shah JL, Iyer SN, Lal S: **Transforming youth mental health care in a semi-urban and rural region of Canada: A service description of ACCESS Open Minds Chatham-Kent**. *Early Interv Psychiatry* 2019, **13 Suppl 1**:48-55.

94. Robinson LJ, Stephens NM, Wilson S, Graham L, Hackett KL: **Conceptualizing the key components of rehabilitation following major musculoskeletal trauma: A mixed methods service evaluation**. *J Eval Clin Pract* 2019:1-12.

95. Romm KL, Gardsjord ES, Gjermundsen K, Ulloa MA, Berentzen LC, Melle I: **Designing easy access to care for first-episode psychosis in complex organizations**. *Early Interv Psychiatry* 2019, **13**(5):1276-1282.

96. Ruland CM, Starren J, Vatne TM: **Participatory design with children in the development of a support system for patient-centered care in pediatric oncology**. *J Biomed Inform* 2008, **41**(4):624-635.

97. Tsimicalis A, Stone PW, Bakken S, Yoon S, Sands S, Porter R, Ruland C: **Usability testing of a computerized communication tool in a diverse urban pediatric population**. *Cancer Nurs* 2014, **37**(6):E25-E34.

98. Arvidsson S, Gilljam B-M, Nygren J, Ruland CM, Nordby-Bøe T, Svedberg P: **Redesign and validation of Sisom, an interactive assessment and communication tool for children with cancer**. In: *JMIR Mhealth Uhealth.* vol. 4; 2016: e76.

99. Tsimicalis A, Le May S, Stinson J, Rennick J, Vachon M-F, Louli J, Bérubé S, Treherne S, Yoon S, Nordby Bøe T: **Linguistic validation of an interactive communication tool to help French-speaking children express their cancer symptoms**. *J Pediatr Oncol Nurs* 2017, **34**(2):98-105.

100. Tsimicalis A, Rennick J, Stinson J, May SL, Louli J, Choquette A, Treherne S, Berube S, Yoon S, Ruland C: **Usability Testing of an Interactive Communication Tool to Help Children Express Their Cancer Symptoms**. *J Pediatr Oncol Nurs* 2018, **35**(5):320-331.

101. Taylor J, Coates E, Wessels B, Mountain G, Hawley MS: **Implementing solutions to improve and expand telehealth adoption: participatory action research in four community healthcare settings**. In: *BMC Health Serv Res.* vol. 15; 2015.

102. Thomson A, Rivas C, Giovannoni G: **Multiple sclerosis outpatient future groups: improving the quality of participant interaction and ideation tools within service improvement activities**. In: *BMC Health Serv Res.* vol. 15; 2015.

103. Tsianakas V, Robert G, Maben J, Richardson A, Dale C, Wiseman T: **Implementing patient-centred cancer care: using experience-based co-design to improve patient experience in breast and lung cancer services**. *Support Care Cancer* 2012, **20**(11):2639-2647.

104. Valaitis R, Longaphy J, Ploeg J, Agarwal G, Oliver D, Nair K, Kastner M, Avilla E, Dolovich L: **Health TAPESTRY: co-designing interprofessional primary care programs for older adults using the persona-scenario method**. *BMC Fam Pract* 2019, **20**(1):122.

105. Wärnestål P, Svedberg P, Lindberg S, Nygren JM: **Effects of using child personas in the development of a digital peer support service for childhood cancer survivors**. *J Med Internet Res* 2017, **19**(5):e161.

106. Woods L, Cummings E, Duff J, Walker K: **Conceptual Design and Iterative Development of a mHealth App by Clinicians, Patients and Their Families**. *Stud Health Technol Inform* 2018, **252**:170-175.

107. Woods LS, Duff J, Roehrer E, Walker K, Cummings E: **Patients’ Experiences of Using a Consumer mHealth App for Self-Management of Heart Failure: Mixed-Methods Study**. *JMIR Human Factors* 2019, **6**(2).

108. Woods L, Roehrer E, Duff J, Walker K, Cummings E: **Co-Design of a Mobile Health App for Heart Failure: Perspectives from the Team**. In: *Digital Health: Changing the Way Healthcare is Conceptualised and Delivered: Selected Papers from the 27th Australian National Health Informatics Conference (HIC 2019): 2019*: IOS Press; 2019: 183.

109. Yu CH, Ke C, Jovicic A, Hall S, Straus SE: **Beyond pros and cons - developing a patient decision aid to cultivate dialog to build relationships: insights from a qualitative study and decision aid development**. *BMC Med Inform Decis Mak* 2019, **19**(1):186.
