# Supplementary material for: Evaluating health service outcomes of public involvement in health service design in high-income countries: a systematic review
Source: BMC Health Serv Res. 2021 Apr 20;21:364. doi: 10.1186/s12913-021-06319-1 (PMC8056601; doi:10.1186/s12913-021-06319-1)
Supplement: Supplementary file 3 — Additional file 3: Quality apprisals of included studies. [file 12913_2021_6319_MOESM3_ESM.docx]

**Additional file 3: Quality appraisals of included studies**

**Table S1: Quality appraisal as assessed by the Mixed Methods Appraisal Tool (MMAT).**

| **Primary author, year** | **Screening questions** | | **Criterion** | | | | | | | | | |
| --- | --- | --- | --- | --- | --- | --- | --- | --- | --- | --- | --- | --- |
|  | S1 | S2 | 1.1 | 1.2 | 1.3 | 1.4 | 1.5 | 3.1 | 3.2 | 3.3 | 3.4 | 3.5 |
| Adamou 2016 [1] | Can’t tell | Can’t tell |  |  |  |  |  |  |  |  |  |  |
| Airoldi 2013 [2] | X | Can’t tell |  |  |  |  |  |  |  |  |  |  |
| Baggott 2015 [3] | X | Can’t tell |  |  |  |  |  |  |  |  |  |  |
| Bauer 2018 [4] | X | Can’t tell |  |  |  |  |  |  |  |  |  |  |
| Beauchamp 2017 [5] | X | Can’t tell |  |  |  |  |  |  |  |  |  |  |
| Blanco 2019 [6] | X | Can’t tell |  |  |  |  |  |  |  |  |  |  |
| Boaz 2016 [7] | ✓ | ✓ | ✓ | ✓ | ✓ | ✓ | ✓ |  |  |  |  |  |
| Borosund 2018 [8] | X | Can’t tell |  |  |  |  |  |  |  |  |  |  |
| Boyd 2012 [9] | X | Can’t tell |  |  |  |  |  |  |  |  |  |  |
| Burbach 2019 [10] | X | Can’t tell |  |  |  |  |  |  |  |  |  |  |
| Calvillo-Arbizu 2019 [11] | X | Can’t tell |  |  |  |  |  |  |  |  |  |  |
| Castensoe-Seidenfaden 2017 [12] | X | Can’t tell |  |  |  |  |  |  |  |  |  |  |
| Chapman 2018 [13] | ✓ | ✓ |  |  |  |  |  | ✓ | ✓ | ✓ | ✓ | ✓ |
| Chappel 2001 [14] | X | Can’t tell |  |  |  |  |  |  |  |  |  |  |
| Cheng 2011 [15] | X | Can’t tell |  |  |  |  |  |  |  |  |  |  |
| Coad 2008 [16] | ✓ | ✓ | ✓ | ✓ | ✓ | ✓ | ✓ |  |  |  |  |  |
| Collins 2017 [17] | X | Can’t tell |  |  |  |  |  |  |  |  |  |  |
| Cook 2007 [18] | X | Can’t tell |  |  |  |  |  |  |  |  |  |  |
| Cook 2010 [19] | X | Can’t tell |  |  |  |  |  |  |  |  |  |  |
| Cooke 2014 [20] | ✓ | ✓ | ✓ | ✓ | ✓ | X | Can’t tell |  |  |  |  |  |
| Cooper 2016 [21] | X | Can’t tell |  |  |  |  |  |  |  |  |  |  |
| Cotterell 2004 [22] | X | Can’t tell |  |  |  |  |  |  |  |  |  |  |
| Coylewright 2012 [23] | X | Can’t tell |  |  |  |  |  |  |  |  |  |  |
| Cramp 2006 [24] | X | Can’t tell |  |  |  |  |  |  |  |  |  |  |
| Crowley 2002 [25] | ✓ | ✓ | ✓ | ✓ | ✓ | ✓ | ✓ |  |  |  |  |  |
| Csipke 2016 [26] | X | Can’t tell |  |  |  |  |  |  |  |  |  |  |
| Cushen 2004 [27] | X | Can’t tell |  |  |  |  |  |  |  |  |  |  |
| Das 2013 [28] | X | Can’t tell |  |  |  |  |  |  |  |  |  |  |
| de Souza 2017 [29] | X | Can’t tell |  |  |  |  |  |  |  |  |  |  |
| Dewar 2010 [30] | X | Can’t tell |  |  |  |  |  |  |  |  |  |  |
| Diamond 2003 [31] | X | Can’t tell |  |  |  |  |  |  |  |  |  |  |
| Dinniss 2007 [32] | X | Can’t tell |  |  |  |  |  |  |  |  |  |  |
| Doherty 2018 [33] | X | Can’t tell |  |  |  |  |  |  |  |  |  |  |
| Dorrington 2015 [34] | X | Can’t tell |  |  |  |  |  |  |  |  |  |  |
| Douglas 2005 [35] | X | Can’t tell |  |  |  |  |  |  |  |  |  |  |
| Doyle 2016 [36] | X | Can’t tell |  |  |  |  |  |  |  |  |  |  |
| Durey 2016 [37] | X | Can’t tell |  |  |  |  |  |  |  |  |  |  |
| Edwards 2016 [38] | X | Can’t tell |  |  |  |  |  |  |  |  |  |  |
| Ennis 2014 [39] | X | Can’t tell |  |  |  |  |  |  |  |  |  |  |
| Farr 2019 [40] | X | Can’t tell |  |  |  |  |  |  |  |  |  |  |
| Fitzgerald 2011 [41] | X | Can’t tell |  |  |  |  |  |  |  |  |  |  |
| Forchuk 1998 [42] | X | Can’t tell |  |  |  |  |  |  |  |  |  |  |
| Gardener 2019 [43] | X | Can’t tell |  |  |  |  |  |  |  |  |  |  |
| Hahn-Goldberg 2015 [44] | X | Can’t tell |  |  |  |  |  |  |  |  |  |  |
| Hahn-Goldberg 2016 [45] | X | Can’t tell |  |  |  |  |  |  |  |  |  |  |
| Han 2018 [46] | Can’t tell | X |  |  |  |  |  |  |  |  |  |  |
| Hickman 2019 [47] | Can’t tell | X |  |  |  |  |  |  |  |  |  |  |
| Hobson 2018 [48] | Can’t tell | X |  |  |  |  |  |  |  |  |  |  |
| Holloway 2006 [49] | X | Can’t tell |  |  |  |  |  |  |  |  |  |  |
| Irving 2018 [50] | X | Can’t tell |  |  |  |  |  |  |  |  |  |  |
| Isenberg 2018 [51] | X | Can’t tell |  |  |  |  |  |  |  |  |  |  |
| Jackson 2003 [52] | X | Can’t tell |  |  |  |  |  |  |  |  |  |  |
| Jessup 2018 [53] | ✓ | ✓ | ✓ | ✓ | ✓ | ✓ | ✓ |  |  |  |  |  |
| Jones 2008 [54] | X | Can’t tell |  |  |  |  |  |  |  |  |  |  |
| Kennedy 2014 [55] | X | Can’t tell |  |  |  |  |  |  |  |  |  |  |
| Kenyon 2016 [56] | X | Can’t tell |  |  |  |  |  |  |  |  |  |  |
| Kidd 2015 [57] | X | Can’t tell |  |  |  |  |  |  |  |  |  |  |
| Kilander 2019 [58] | X | Can’t tell |  |  |  |  |  |  |  |  |  |  |
| Kildea 2018 [59] | X | Can’t tell |  |  |  |  |  |  |  |  |  |  |
| Knight 2007 [60] | X | Can’t tell |  |  |  |  |  |  |  |  |  |  |
| Kohler 2017 [61] | X | Can’t tell |  |  |  |  |  |  |  |  |  |  |
| Krist 2011 [62] | X | Can’t tell |  |  |  |  |  |  |  |  |  |  |
| Larkin 2015 [63] | X | Can’t tell |  |  |  |  |  |  |  |  |  |  |
| Latif 2017 [64] | X | Can’t tell |  |  |  |  |  |  |  |  |  |  |
| Lo 2018 [65] | X | Can’t tell |  |  |  |  |  |  |  |  |  |  |
| Locock 2014 [66] | ✓ | ✓ | ✓ | ✓ | ✓ | ✓ | ✓ |  |  |  |  |  |
| Lopatina 2019 [67] | X | Can’t tell |  |  |  |  |  |  |  |  |  |  |
| Lyles 2016 [68] | X | Can’t tell |  |  |  |  |  |  |  |  |  |  |
| Manning 2017 [69] | X | Can’t tell |  |  |  |  |  |  |  |  |  |  |
| Marshall 2006 [70] | X | Can’t tell |  |  |  |  |  |  |  |  |  |  |
| McClelland 2018 [71] | X | Can’t tell |  |  |  |  |  |  |  |  |  |  |
| McWilliams 2018 [72] | X | Can’t tell |  |  |  |  |  |  |  |  |  |  |
| Meldrum 2006 [73] | X | Can’t tell |  |  |  |  |  |  |  |  |  |  |
| Melnick 2017 [74] | X | Can’t tell |  |  |  |  |  |  |  |  |  |  |
| Olding 2018 [75] | X | Can’t tell |  |  |  |  |  |  |  |  |  |  |
| Outlaw 2018 [76] | X | Can’t tell |  |  |  |  |  |  |  |  |  |  |
| Owens 2011 [77] | X | Can’t tell |  |  |  |  |  |  |  |  |  |  |
| Pilgrim 1998 [78] | X | Can’t tell |  |  |  |  |  |  |  |  |  |  |
| Piper 2012 [79] | X | Can’t tell |  |  |  |  |  |  |  |  |  |  |
| Powell 1994 [80] | X | Can’t tell |  |  |  |  |  |  |  |  |  |  |
| Probst 2018 [81] | X | Can’t tell |  |  |  |  |  |  |  |  |  |  |
| Reaume-Zimmer 2019 [82] | X | Can’t tell |  |  |  |  |  |  |  |  |  |  |
| Robinson 2019 [83] | X | Can’t tell |  |  |  |  |  |  |  |  |  |  |
| Romm 2019 [84] | X | Can’t tell |  |  |  |  |  |  |  |  |  |  |
| Ruland 2008 [85] | X | Can’t tell |  |  |  |  |  |  |  |  |  |  |
| Taylor 2015 [86] | X | Can’t tell |  |  |  |  |  |  |  |  |  |  |
| Thomson 2015 [87] | X | Can’t tell |  |  |  |  |  |  |  |  |  |  |
| Tsianakas 2012 [88] | X | Can’t tell |  |  |  |  |  |  |  |  |  |  |
| Tsimicalis 2018 [89] | X | Can’t tell |  |  |  |  |  |  |  |  |  |  |
| Valaitis 2019 [90] | X | Can’t tell |  |  |  |  |  |  |  |  |  |  |
| Warnestal 2017 [91] | X | Can’t tell |  |  |  |  |  |  |  |  |  |  |
| Woods 2018 [92] | X | Can’t tell |  |  |  |  |  |  |  |  |  |  |
| Yu 2019 [93] | X | Can’t tell |  |  |  |  |  |  |  |  |  |  |

**Abbreviations**: ✓Yes, X No.

MMAT Quality Appraisal Criteria [94]:

Screening questions:

S1. Are there clear research questions?

S2. Do the collected data allow to address the research questions?

Qualitative

1.1 Is the qualitative approach appropriate to answer the research question?

1.2 Are the qualitative data collection methods adequate to address the research question?

1.3 Are the findings adequately derived from the data?

1.4 Is the interpretation of results sufficiently substantiated by data?

1.5 Is there coherence between qualitative data sources, collection, analysis and interpretation?

Non-randomized studies

3.1 Are the participants representative of the target population?

3.2 Are measurements appropriate regarding both the outcome and intervention (or exposure)?

3.3. Are there complete outcome data?

3.4 Are the confounders accounted for in the design and analysis?

3.5 During the study period, is the intervention administered (or exposure occurred) as intended?

**Table S2: Critical appraisal guidelines**

| \|  \| **Author/Year** \| 1 \| 2 \| 3 \| 4 \| 5 \| 6 \| 7 \| 8 \| 9 \| **Total criteria met** \| \| --- \| --- \| --- \| --- \| --- \| --- \| --- \| --- \| --- \| --- \| --- \| --- \| \|  \| Adamou 2016 [1] \|  \|  \| ✓ \|  \|  \|  \|  \|  \|  \| 1 \| \|  \| Airoldi 2013 [2] \|  \|  \|  \|  \|  \|  \|  \| ✓ \|  \| 1 \| \|  \| Baggott 2015 [3] \| ✓ \| ✓ \| ✓ \|  \| ✓ \| ✓ \|  \|  \|  \| 5 \| \|  \| Bauer 2018 [4] \| ✓ \| ✓ \|  \|  \|  \|  \|  \| ✓ \|  \| 3 \| \|  \| Beauchamp 2017 [5] \|  \| ✓ \|  \|  \|  \|  \|  \| ✓ \| ✓ \| 3 \| \|  \| Blanco 2019 [6] \| ✓ \| ✓ \|  \|  \|  \|  \|  \|  \|  \| 2 \| \|  \| Boaz 2016 [7] \| ✓ \| ✓ \|  \|  \| ✓ \| ✓ \| ✓ \| ✓ \| ✓ \| 7 \| \|  \| Borosund 2018 [8] \| ✓ \| ✓ \| ✓ \|  \|  \|  \|  \| ✓ \|  \| 4 \| \|  \| Boyd 2012 [9] \| ✓ \| ✓ \|  \|  \|  \|  \|  \| ✓ \|  \| 3 \| \|  \| Burbach 2019 [10] \|  \| ✓ \|  \| ✓ \|  \|  \|  \| ✓ \| ✓ \| 4 \| \|  \| Calvillo-Arbizu 2019 [11] \| ✓ \| ✓ \|  \|  \|  \|  \|  \| ✓ \|  \| 3 \| \|  \| Castesoe-Seidefaden 2017 [12] \| ✓ \| ✓ \|  \|  \|  \| ✓ \|  \| ✓ \|  \| 4 \| \|  \| Chapman 2018 [13] \|  \|  \|  \|  \|  \|  \|  \|  \|  \| 0 \| \|  \| Chappel 2001 [14] \| ✓ \| ✓ \| ✓ \|  \|  \|  \|  \|  \|  \| 3 \| \|  \| Cheng 2011 [15] \|  \| ✓ \|  \|  \|  \|  \|  \|  \|  \| 1 \| \|  \| Coad 2008 [16] \| ✓ \| ✓ \| ✓ \|  \| ✓ \| ✓ \| ✓ \| ✓ \| ✓ \| 8 \| \|  \| Collins 2017 [17] \| ✓ \| ✓ \|  \|  \| ✓ \|  \|  \| ✓ \|  \| 4 \| \|  \| Cook 2007 [18] \| ✓ \|  \|  \|  \|  \|  \|  \|  \|  \| 1 \| \|  \| Cook 2010 [19] \| ✓ \| ✓ \|  \|  \|  \|  \|  \|  \|  \| 2 \| \|  \| Cooke 2014 [20] \| ✓ \| ✓ \| ✓ \|  \| ✓ \|  \|  \| ✓ \|  \| 5 \| \|  \| Cooper 2016 [21] \| ✓ \| ✓ \| ✓ \| ✓ \| ✓ \| ✓ \|  \|  \| ✓ \| 7 \| \|  \| Cotterell 2004 [22] \| ✓ \|  \|  \|  \| ✓ \|  \|  \| ✓ \| ✓ \| 4 \| \|  \| Coylewright 2012 [23] \| ✓ \| ✓ \|  \|  \|  \|  \|  \| ✓ \|  \| 3 \| \|  \| Cramp 2006 [24] \| ✓ \| ✓ \|  \|  \|  \|  \|  \|  \|  \| 2 \| \|  \| Crowley 2002 [25] \| ✓ \| ✓ \|  \|  \|  \|  \|  \| ✓ \| ✓ \| 4 \| \|  \| Csipke 2016 [26] \| ✓ \| ✓ \| ✓ \|  \|  \|  \|  \| ✓ \|  \| 4 \| \|  \| Cushen 2004 [27] \| ✓ \| ✓ \| ✓ \|  \|  \| ✓ \|  \| ✓ \| ✓ \| 6 \| \|  \| Das 2013 [28] \| ✓ \| ✓ \| ✓ \|  \| ✓ \| ✓ \|  \| ✓ \| ✓ \| 7 \| \|  \| de Souza 2017 [29] \| ✓ \| ✓ \| ✓ \|  \|  \| ✓ \|  \| ✓ \|  \| 5 \| \|  \| Dewar 2010 [30] \| ✓ \| ✓ \| ✓ \|  \| ✓ \| ✓ \|  \| ✓ \| ✓ \| 7 \| \|  \| Diamond 2003 [31] \| ✓ \| ✓ \|  \|  \|  \|  \|  \| ✓ \| ✓ \| 4 \| \|  \| Dinniss 2007 [32] \| ✓ \| ✓ \| ✓ \|  \|  \| ✓ \|  \| ✓ \|  \| 5 \| \|  \| Doherty 2018 [33] \| ✓ \| ✓ \| ✓ \|  \|  \|  \|  \| ✓ \|  \| 4 \| \|  \| Dorrington 2015 [34] \| ✓ \| ✓ \| ✓ \|  \| ✓ \|  \|  \| ✓ \|  \| 5 \| \|  \| Douglas 2005 [35] \| ✓ \| ✓ \| ✓ \|  \|  \|  \|  \| ✓ \|  \| 4 \| \|  \| Doyle 2016 [36] \|  \|  \|  \| ✓ \|  \|  \|  \| ✓ \|  \| 2 \| \|  \| Durey 2016 [37] \| ✓ \| ✓ \| ✓ \| ✓ \|  \|  \|  \| ✓ \| ✓ \| 6 \| \|  \| Edwards 2016 [38] \| ✓ \| ✓ \| ✓ \|  \|  \| ✓ \| ✓ \| ✓ \|  \| 6 \| \|  \| Ennis 2014 [39] \|  \| ✓ \|  \|  \|  \|  \|  \| ✓ \|  \| 2 \| \|  \| Farr 2019 [40] \| ✓ \| ✓ \|  \|  \|  \|  \|  \| ✓ \|  \| 3 \| \|  \| Fitzgerald 2011 [41] \| ✓ \| ✓ \| ✓ \|  \|  \|  \|  \| ✓ \|  \| 4 \| \|  \| Forchuk 1998 [42] \|  \| ✓ \|  \|  \|  \|  \|  \|  \|  \| 1 \| \|  \| Gardener 2019 [43] \| ✓ \| ✓ \| ✓ \|  \| ✓ \|  \|  \| ✓ \|  \| 5 \| \|  \| Hahn-Goldberg 2015 [44] \| ✓ \| ✓ \|  \|  \|  \|  \|  \| ✓ \|  \| 3 \| \|  \| Hahn-Goldberg 2016 [45] \| ✓ \| ✓ \|  \|  \|  \|  \|  \| ✓ \|  \| 3 \| \|  \| Han 2018 [46] \| ✓ \| ✓ \|  \|  \|  \|  \|  \|  \|  \| 2 \| \|  \| Hickman 2019 [47] \| ✓ \| ✓ \| ✓ \|  \|  \| ✓ \|  \| ✓ \|  \| 5 \| \|  \| Hobson 2018 [48] \| ✓ \| ✓ \| ✓ \|  \| ✓ \|  \|  \| ✓ \| ✓ \| 6 \| \|  \| Holloway 2006 [49] \|  \| ✓ \|  \|  \|  \|  \|  \|  \|  \| 1 \| \|  \| Irving 2018 [50] \| ✓ \| ✓ \| ✓ \|  \| ✓ \| ✓ \| ✓ \| ✓ \| ✓ \| 8 \| \|  \| Isenberg 2018 [51] \| ✓ \| ✓ \|  \|  \|  \|  \| ✓ \| ✓ \| ✓ \| 5 \| \|  \| Jackson 2003 [52] \| ✓ \| ✓ \| ✓ \| ✓ \|  \| ✓ \|  \| ✓ \| ✓ \| 7 \| \|  \| Jessup 2018 [53] \| ✓ \| ✓ \|  \|  \|  \|  \|  \| ✓ \|  \| 3 \| \|  \| Jones 2008 [54] \| ✓ \| ✓ \| ✓ \|  \| ✓ \|  \|  \| ✓ \|  \| 5 \| \|  \| Kennedy 2014 [55] \| ✓ \| ✓ \| ✓ \|  \|  \|  \|  \| ✓ \|  \| 4 \| \|  \| Kenyon 2016 [56] \| ✓ \| ✓ \| ✓ \|  \| ✓ \|  \|  \| ✓ \|  \| 4 \| \|  \| Kidd 2015 [57] \| ✓ \| ✓ \| ✓ \|  \|  \|  \| ✓ \| ✓ \|  \| 5 \| \|  \| Kilander 2019 [58] \|  \| ✓ \|  \|  \|  \|  \|  \|  \|  \| 1 \| \|  \| Kildea 2018 [59] \|  \| ✓ \|  \|  \|  \|  \|  \|  \|  \| 1 \| \|  \| Knight 2007 [60] \| ✓ \| ✓ \| ✓ \|  \| ✓ \|  \|  \|  \|  \| 4 \| \|  \| Kohler 2017 [61] \| ✓ \| ✓ \|  \|  \|  \|  \|  \| ✓ \|  \| 3 \| \|  \| Krist 2011 [62] \| ✓ \| ✓ \|  \|  \|  \|  \|  \|  \|  \| 2 \| \|  \| Larkin 2015 [63] \| ✓ \| ✓ \|  \|  \| ✓ \| ✓ \|  \| ✓ \| ✓ \| 6 \| \|  \| Latif 2017 [64] \| ✓ \| ✓ \| ✓ \|  \| ✓ \|  \|  \| ✓ \| ✓ \| 6 \| \|  \| Lo 2018 [65] \|  \|  \|  \|  \|  \|  \|  \| ✓ \|  \| 1 \| \|  \| Locock 2014 [66] \| ✓ \| ✓ \|  \|  \|  \|  \|  \| ✓ \| ✓ \| 4 \| \|  \| Lopatina 2019 [67] \| ✓ \| ✓ \|  \| ✓ \|  \| ✓ \|  \| ✓ \|  \| 5 \| \|  \| Lyles 2016 [68] \| ✓ \| ✓ \| ✓ \|  \|  \|  \|  \| ✓ \| ✓ \| 5 \| \|  \| Manning 2017 [69] \| ✓ \| ✓ \| ✓ \|  \|  \|  \|  \| ✓ \|  \| 4 \| \|  \| Marshall 2006 [70] \| ✓ \| ✓ \| ✓ \|  \|  \|  \|  \| ✓ \|  \| 4 \| \|  \| McClelland 2018 [71] \| ✓ \| ✓ \| ✓ \|  \|  \|  \|  \| ✓ \|  \| 4 \| \|  \| McWilliams 2018 [72] \| ✓ \| ✓ \| ✓ \|  \| ✓ \|  \|  \| ✓ \|  \| 5 \| \|  \| Meldrum 2006 [73] \| ✓ \| ✓ \|  \|  \|  \|  \|  \| ✓ \|  \| 3 \| \|  \| Melnick 2017 [74] \| ✓ \| ✓ \| ✓ \|  \| ✓ \| ✓ \|  \| ✓ \| ✓ \| 7 \| \|  \| Olding 2018 [75] \| ✓ \| ✓ \|  \|  \|  \|  \|  \| ✓ \|  \| 3 \| \|  \| Outlaw 2018 [76] \| ✓ \| ✓ \| ✓ \|  \|  \| ✓ \| ✓ \| ✓ \|  \| 6 \| \|  \| Owens 2011 [77] \| ✓ \| ✓ \| ✓ \|  \|  \|  \|  \|  \| ✓ \| 4 \| \|  \| Pilgrim 1998 [78] \| ✓ \| ✓ \| ✓ \|  \|  \|  \|  \|  \|  \| 3 \| \|  \| Piper 2012 [79] \| ✓ \| ✓ \| ✓ \|  \|  \|  \|  \| ✓ \|  \| 4 \| \|  \| Powell 1994 [80] \| ✓ \| ✓ \| ✓ \|  \|  \|  \|  \| ✓ \|  \| 4 \| \|  \| Probst 2018 [81] \| ✓ \| ✓ \|  \|  \|  \|  \|  \| ✓ \| ✓ \| 4 \| \|  \| Reaume-Zimmer 2019 [82] \| ✓ \| ✓ \| ✓ \|  \|  \| ✓ \|  \| ✓ \|  \| 5 \| \|  \| Robison 2019 [83] \| ✓ \| ✓ \|  \|  \|  \|  \|  \| ✓ \| ✓ \| 4 \| \|  \| Romm 2019 [84] \| ✓ \| ✓ \| ✓ \|  \| ✓ \| ✓ \|  \| ✓ \|  \| 6 \| \|  \| Ruland 2008 [85] \| ✓ \| ✓ \| ✓ \|  \|  \| ✓ \|  \|  \|  \| 4 \| \|  \| Taylor 2015 [86] \| ✓ \| ✓ \| ✓ \|  \| ✓ \|  \|  \| ✓ \|  \| 5 \| \|  \| Thomson 2015 [87] \| ✓ \| ✓ \| ✓ \|  \|  \|  \|  \| ✓ \| ✓ \| 5 \| \|  \| Tsianakas 2012 [88] \| ✓ \| ✓ \| ✓ \|  \| ✓ \|  \|  \| ✓ \|  \| 4 \| \|  \| Tsimicalis 2018 [89] \| ✓ \| ✓ \| ✓ \|  \|  \| ✓ \|  \| ✓ \| ✓ \| 5 \| \|  \| Valaitis 2019 [90] \| ✓ \| ✓ \| ✓ \|  \| ✓ \|  \|  \| ✓ \|  \| 5 \| \|  \| Warnestal 2017 [91] \| ✓ \|  \|  \|  \|  \|  \|  \|  \|  \| 1 \| \|  \| Woods 2018 [92] \| ✓ \| ✓ \|  \|  \|  \|  \|  \| ✓ \|  \| 3 \| \|  \| Yu 2019 [93] \|  \|  \|  \|  \|  \|  \|  \|  \|  \|  \| \|  \|  \|  \|  \|  \|  \|  \|  \|  \|  \|  \|  \| \|  \| % of articles achieving criterion \| 83.9% \| 90/3% \| 52.7% \| 6.5% \| 25.8% \| 23.7% \| 7.5% \| 76.3% \| 28.0% \|  \| \|  \|  \|  \|  \|  \|  \|  \|  \|  \|  \|  \|  \| |
| --- | --- | --- | --- | --- | --- | --- | --- | --- | --- | --- | --- | --- | --- | --- | --- | --- | --- | --- | --- | --- | --- | --- | --- | --- | --- | --- | --- | --- | --- | --- | --- | --- | --- | --- | --- | --- | --- | --- | --- | --- | --- | --- | --- | --- | --- | --- | --- | --- | --- | --- | --- | --- | --- | --- | --- | --- | --- | --- | --- | --- | --- | --- | --- | --- | --- | --- | --- | --- | --- | --- | --- | --- | --- | --- | --- | --- | --- | --- | --- | --- | --- | --- | --- | --- | --- | --- | --- | --- | --- | --- | --- | --- | --- | --- | --- | --- | --- | --- | --- | --- | --- | --- | --- | --- | --- | --- | --- | --- | --- | --- | --- | --- | --- | --- | --- | --- | --- | --- | --- | --- | --- | --- | --- | --- | --- | --- | --- | --- | --- | --- | --- | --- | --- | --- | --- | --- | --- | --- | --- | --- | --- | --- | --- | --- | --- | --- | --- | --- | --- | --- | --- | --- | --- | --- | --- | --- | --- | --- | --- | --- | --- | --- | --- | --- | --- | --- | --- | --- | --- | --- | --- | --- | --- | --- | --- | --- | --- | --- | --- | --- | --- | --- | --- | --- | --- | --- | --- | --- | --- | --- | --- | --- | --- | --- | --- | --- | --- | --- | --- | --- | --- | --- | --- | --- | --- | --- | --- | --- | --- | --- | --- | --- | --- | --- | --- | --- | --- | --- | --- | --- | --- | --- | --- | --- | --- | --- | --- | --- | --- | --- | --- | --- | --- | --- | --- | --- | --- | --- | --- | --- | --- | --- | --- | --- | --- | --- | --- | --- | --- | --- | --- | --- | --- | --- | --- | --- | --- | --- | --- | --- | --- | --- | --- | --- | --- | --- | --- | --- | --- | --- | --- | --- | --- | --- | --- | --- | --- | --- | --- | --- | --- | --- | --- | --- | --- | --- | --- | --- | --- | --- | --- | --- | --- | --- | --- | --- | --- | --- | --- | --- | --- | --- | --- | --- | --- | --- | --- | --- | --- | --- | --- | --- | --- | --- | --- | --- | --- | --- | --- | --- | --- | --- | --- | --- | --- | --- | --- | --- | --- | --- | --- | --- | --- | --- | --- | --- | --- | --- | --- | --- | --- | --- | --- | --- | --- | --- | --- | --- | --- | --- | --- | --- | --- | --- | --- | --- | --- | --- | --- | --- | --- | --- | --- | --- | --- | --- | --- | --- | --- | --- | --- | --- | --- | --- | --- | --- | --- | --- | --- | --- | --- | --- | --- | --- | --- | --- | --- | --- | --- | --- | --- | --- | --- | --- | --- | --- | --- | --- | --- | --- | --- | --- | --- | --- | --- | --- | --- | --- | --- | --- | --- | --- | --- | --- | --- | --- | --- | --- | --- | --- | --- | --- | --- | --- | --- | --- | --- | --- | --- | --- | --- | --- | --- | --- | --- | --- | --- | --- | --- | --- | --- | --- | --- | --- | --- | --- | --- | --- | --- | --- | --- | --- | --- | --- | --- | --- | --- | --- | --- | --- | --- | --- | --- | --- | --- | --- | --- | --- | --- | --- | --- | --- | --- | --- | --- | --- | --- | --- | --- | --- | --- | --- | --- | --- | --- | --- | --- | --- | --- | --- | --- | --- | --- | --- | --- | --- | --- | --- | --- | --- | --- | --- | --- | --- | --- | --- | --- | --- | --- | --- | --- | --- | --- | --- | --- | --- | --- | --- | --- | --- | --- | --- | --- | --- | --- | --- | --- | --- | --- | --- | --- | --- | --- | --- | --- | --- | --- | --- | --- | --- | --- | --- | --- | --- | --- | --- | --- | --- | --- | --- | --- | --- | --- | --- | --- | --- | --- | --- | --- | --- | --- | --- | --- | --- | --- | --- | --- | --- | --- | --- | --- | --- | --- | --- | --- | --- | --- | --- | --- | --- | --- | --- | --- | --- | --- | --- | --- | --- | --- | --- | --- | --- | --- | --- | --- | --- | --- | --- | --- | --- | --- | --- | --- | --- | --- | --- | --- | --- | --- | --- | --- | --- | --- | --- | --- | --- | --- | --- | --- | --- | --- | --- | --- | --- | --- | --- | --- | --- | --- | --- | --- | --- | --- | --- | --- | --- | --- | --- | --- | --- | --- | --- | --- | --- | --- | --- | --- | --- | --- | --- | --- | --- | --- | --- | --- | --- | --- | --- | --- | --- | --- | --- | --- | --- | --- | --- | --- | --- | --- | --- | --- | --- | --- | --- | --- | --- | --- | --- | --- | --- | --- | --- | --- | --- | --- | --- | --- | --- | --- | --- | --- | --- | --- | --- | --- | --- | --- | --- | --- | --- | --- | --- | --- | --- | --- | --- | --- | --- | --- | --- | --- | --- | --- | --- | --- | --- | --- | --- | --- | --- | --- | --- | --- | --- | --- | --- | --- | --- | --- | --- | --- | --- | --- | --- | --- | --- | --- | --- | --- | --- | --- | --- | --- | --- | --- | --- | --- | --- | --- | --- | --- | --- | --- | --- | --- | --- | --- | --- | --- | --- | --- | --- | --- | --- | --- | --- | --- | --- | --- | --- | --- | --- | --- | --- | --- | --- | --- | --- | --- | --- | --- | --- | --- | --- | --- | --- | --- | --- | --- | --- | --- | --- | --- | --- | --- | --- | --- | --- | --- | --- | --- | --- | --- | --- | --- | --- | --- | --- | --- | --- | --- | --- | --- | --- | --- | --- | --- | --- | --- | --- | --- | --- | --- | --- | --- | --- | --- | --- | --- | --- | --- | --- | --- | --- | --- | --- | --- | --- | --- | --- | --- | --- | --- | --- | --- | --- | --- | --- | --- | --- | --- | --- | --- | --- | --- | --- | --- | --- | --- | --- | --- | --- | --- | --- | --- | --- | --- | --- | --- | --- | --- | --- | --- | --- | --- | --- | --- | --- | --- | --- | --- | --- | --- | --- | --- | --- | --- | --- | --- | --- | --- | --- | --- | --- | --- | --- | --- | --- | --- | --- | --- | --- | --- | --- | --- | --- | --- | --- | --- | --- | --- | --- | --- | --- | --- | --- | --- | --- | --- | --- | --- | --- | --- | --- | --- | --- | --- | --- | --- | --- | --- | --- | --- | --- | --- | --- | --- | --- | --- | --- | --- | --- | --- | --- | --- | --- | --- | --- | --- | --- | --- | --- | --- | --- | --- | --- | --- | --- | --- | --- | --- | --- | --- | --- | --- | --- | --- | --- | --- | --- | --- | --- | --- | --- | --- | --- | --- | --- | --- | --- | --- | --- | --- | --- | --- | --- | --- | --- | --- | --- | --- | --- | --- | --- | --- | --- | --- | --- | --- | --- | --- | --- | --- | --- | --- | --- | --- | --- | --- | --- | --- | --- | --- | --- | --- | --- | --- | --- | --- | --- | --- | --- | --- | --- | --- | --- | --- | --- | --- | --- | --- | --- | --- | --- | --- | --- | --- | --- | --- | --- | --- | --- | --- | --- | --- | --- | --- | --- | --- | --- | --- | --- | --- | --- | --- | --- | --- | --- | --- | --- | --- | --- | --- | --- | --- | --- | --- | --- | --- | --- | --- | --- | --- | --- | --- | --- | --- | --- | --- | --- | --- | --- | --- | --- | --- | --- | --- | --- | --- | --- | --- | --- | --- | --- | --- | --- | --- | --- | --- | --- | --- | --- | --- | --- | --- | --- | --- | --- | --- | --- | --- | --- | --- | --- | --- | --- | --- | --- | --- | --- | --- | --- | --- | --- | --- | --- | --- | --- | --- | --- | --- | --- | --- | --- | --- | --- | --- | --- | --- | --- | --- | --- | --- | --- | --- | --- | --- | --- | --- | --- | --- | --- | --- | --- | --- | --- | --- | --- | --- | --- | --- | --- | --- | --- |

Critical appraisal guideline criteria [95]:

1. Is the rationale for involving users clearly demonstrated?
2. Is the level of user involvement appropriate?
3. Is the recruitment strategy appropriate?
4. Is the nature of training appropriate?
5. Has sufficient attention been given to the ethical considerations of user involvement and how these were managed?
6. Has sufficient attention been given to the methodological considerations of user involvement and how these were managed?
7. Have there been any attempts to involve users in the dissemination of findings?
8. Has the ‘added-value’ of user involvement been clearly demonstrated?
9. Have there been any attempts to evaluate the user involvement component of the research?

**References**

1. Adamou M, Graham K, MacKeith J, Burns S, Emerson L-M. Advancing services for adult ADHD: the development of the ADHD Star as a framework for multidisciplinary interventions. BMC Health Serv Res [Internet]. 2016; 16(632). https://doi.org/10.1186/s12913-016-1894-4.

2. Airoldi M. Disinvestments in Practice: Overcoming Resistance to Change through a Sociotechnical Approach with Local Stakeholders. J Health Polit Policy Law. 2013;38(6):1149-71.

3. Baggott C, Baird J, Hinds P, Ruland CM, Miaskowski C. Evaluation of Sisom: A computer-based animated tool to elicit symptoms and psychosocial concerns from children with cancer. Eur J Oncol Nurs. 2015;19(4):359-69.

4. Bauer AM, Hodsdon S, Bechtel JM, Fortney JC. Applying the Principles for Digital Development: Case Study of a Smartphone App to Support Collaborative Care for Rural Patients With Posttraumatic Stress Disorder or Bipolar Disorder. J Med Internet Res. 2018;20(6):e10048.

5. Beauchamp A, Batterham RW, Dodson S, Astbury B, Elsworth GR, McPhee C, et al. Systematic development and implementation of interventions to OPtimise Health Literacy and Access (Ophelia). BMC Public Health [Internet]. 2017; 17(230). https://doi.org/10.1186/s12889-017-4147-5.

6. Blanco T, Casas R, Marco A, Martinez I. Micro ad-hoc Health Social Networks (uHSN). Design and evaluation of a social-based solution for patient support. J Biomed Inform. 2019;89:68-80.

7. Boaz A, Robert G, Locock L, Sturmey G, Gager M, Vougioukalou S, et al. What patients do and their impact on implementation: An ethnographic study of participatory quality improvement projects in English acute hospitals. J Health Organ Manag. 2016;30(2):258-78.

8. Borosund E, Mirkovic J, Clark MM, Ehlers SL, Andrykowski MA, Bergland A, et al. A Stress Management App Intervention for Cancer Survivors: Design, Development, and Usability Testing. JMIR Form Res. 2018;2(2):e19.

9. Boyd H, McKernon S, Mullin B, Old A. Improving healthcare through the use of co-design. N Z Med J. 2012;125(1357):76-87.

10. Burbach FR, Amani SK. Appreciative enquiry peer review improving quality of services. Int J Health Care Qual Assur. 2019;32(5):857-66.

11. Calvillo-Arbizu J, Roa-Romero LM, Estudillo-Valderrama MA, Salgueira-Lazo M, Areste-Fosalba N, del-Castillo-Rodriguez NL, et al. User-centred design for developing e-Health system for renal patients at home (AppNephro). Int J Med Inform. 2019;125:47-54.

12. Castensøe-Seidenfaden P, Husted GR, Teilmann G, Hommel E, Olsen BS, Kensing F. Designing a Self-Management App for Young People With Type 1 Diabetes: Methodological Challenges, Experiences, and Recommendations. JMIR Mhealth Uhealth [Internet]. 2017; 5(10):e124. https://doi.org/10.2196/mhealth.8137.

13. Chapman H, Farndon L, Matthews R, Stephenson J. Okay to Stay? A new plan to help people with long-term conditions remain in their own homes. Prim Health Care Res Dev. 2018;20.

14. Chappel D, Bailey J, Stacy R, Rodgers H, Thomson R. Implementation and evaluation of local-level priority setting for stroke. Public Health. 2001;115(1):21-9.

15. Cheng D, Patel P. Optimizing Women’s Health in a Title X Family Planning Program, Baltimore County, Maryland, 2001-2004. Prev Chronic Dis. 2011;8(6):A126.

16. Coad J, Flay J, Aspinall M, Bilverstone B, Coxhead E, Hones B. Evaluating the impact of involving young people in developing children’s services in an acute hospital trust. J Clin Nurs. 2008;17(23):3115-22.

17. Collins R, Notley C, Clarke T, Wilson J, Fowler D. Participation in developing youth mental health services: “Cinderella service” to service re-design. J Public Ment Health. 2017;16(4):159-68.

18. Cook JA, Ruggiero K, Shore S, Daggett P, Butler SB. Public-academic collaboration in the application of evidence-based practice in Texas mental health system redesign. Int J Ment Health. 2007;36(2):36-49.

19. Cook JA, Shore SE, Burke-Miller JK, Jonikas JA, Ferrara M, Colegrove S, et al. Participatory action research to establish self-directed care for mental health recovery in Texas. Psychiatr Rehabil J. 2010;34(2):137-44.

20. Cooke M, Campbell M. Comparing patient and professional views of expected treatment outcomes for chronic obstructive pulmonary disease: A Delphi study identifies possibilities for change in service delivery in England, UK. J Clin Nurs. 2014;23(13-14):1990-2002.

21. Cooper K, Gillmore C, Hogg L. Experience-based co-design in an adult psychological therapies service. J Ment Health. 2016;25(1):36-40.

22. Cotterell P, Sitzia J, Richardson A. Evaluating partnerships with cancer patients. Practice Nursing. 2004;15(9):430-5.

23. Coylewright M, Shepel K, LeBlanc A, Pencille L, Hess E, Shah N, et al. Shared decision making in patients with stable coronary artery disease: PCI choice. PLoS One. 2012;7(11):e49827.

24. Cramp G. Development of an integrated and sustainable rural service for people with diabetes in the Scottish Highlands. Rural Remote Health. 2006;6(1):422.

25. Crowley P, Green J, Freake D, Drinkwater C. Primary Care Trusts involving the community: is community development the way forward? J Manag Med. 2002;16(4):311-22.

26. Csipke E, Papoulias C, Vitoratou S, Williams P, Rose D, Wykes T. Design in mind: eliciting service user and frontline staff perspectives on psychiatric ward design through participatory methods. J Ment Health. 2016;25(2):114-21.

27. Cushen N, South J, Kruppa S. Patients as teachers: the patient's role in improving cancer services. Prof Nurse. 2004;19(7):395-9.

28. Das A, Svanæs D. Human-centred methods in the design of an e-health solution for patients undergoing weight loss treatment. Int J Med Inform. 2013;82(11):1075-91.

29. de Souza S, Galloway J, Simpson C, Chura R, Dobson J, Gullick NJ, et al. Patient involvement in rheumatology outpatient service design and delivery: a case study. Health Expect. 2017;20(3):508-18.

30. Dewar B, Mackay R, Smith S, Pullin S, Tocher R. Use of emotional touchpoints as a method of tapping into the experience of receiving compassionate care in a hospital setting. J Res Nurs. 2010;15(1):29-41.

31. Diamond B, Parkin G, Morris K, Bettinis J, Bettesworth C. User involvement: substance or spin? J Ment Health. 2003;12(6):613-26.

32. Dinniss S, Roberts G, Hubbard C, Hounsell J, Webb R. User-led assessment of a recovery service using DREEM. Psychiatric Bulletin. 2007;31(4):124-7.

33. Doherty K, Barry M, Marcano-Belisario J, Arnaud B, Morrison C, Car J, et al. A Mobile App for the Self-Report of Psychological Well-Being During Pregnancy (BrightSelf): Qualitative Design Study. JMIR Ment Health. 2018;5(4):e10007.

34. Dorrington MS, Herceg A, Douglas K, Tongs J, Bookallil M. Increasing Pap smear rates at an urban Aboriginal Community Controlled Health Service through translational research and continuous quality improvement. Aust J Prim Health. 2015;21(4):417-22.

35. Douglas CH, Douglas MR. Patient-centred improvements in health-care built environments: perspectives and design indicators. Health Expect. 2005;8(3):264-76.

36. Doyle J, Atkinson-Briggs S, Atkinson P, Firebrace B, Calleja J, Reilly R, et al. A prospective evaluation of first people’s health promotion program design in the goulburn-murray rivers region. BMC Health Serv Res [Internet]. 2016; 16(1):645. https://doi.org/10.1186/s12913-016-1878-4.

37. Durey A, McEvoy S, Swift-Otero V, Taylor K, Katzenellenbogen J, Bessarab D. Improving healthcare for Aboriginal Australians through effective engagement between community and health services. BMC Health Serv Res [Internet]. 2016; 16(224). https://doi.org/10.1186/s12913-016-1497-0.

38. Edwards M, Lawson C, Rahman S, Conley K, Phillips H, Uings R. What does quality healthcare look like to adolescents and young adults? Ask the experts! Clin Med (Lond). 2016;16(2):146-51.

39. Ennis L, Robotham D, Denis M, Pandit N, Newton D, Rose D, et al. Collaborative development of an electronic Personal Health Record for people with severe and enduring mental health problems. BMC Psychiatry. 2014;14(1):305.

40. Farr M, Pithara C, Sullivan S, Edwards H, Hall W, Gadd C, et al. Pilot implementation of co-designed software for co-production in mental health care planning: a qualitative evaluation of staff perspectives. J Ment Health. 2019;28(5):495-504.

41. Fitzgerald MM, Kirk GD, Bristow CA. Description and evaluation of a serious game intervention to engage low secure service users with serious mental illness in the design and refurbishment of their environment. J Psychiatr Ment Health Nurs. 2011;18(4):316-22.

42. Forchuk C, Schofield R, Martin M-L, Sircelj M, Woodcox V, Jewell J, et al. Bridging the discharge process: Staff and client experiences over time. J Am Psychiatr Nurses Assoc. 1998;4(4):128-33.

43. Gardener A, Ewing G, Farquhar M. Enabling patients with advanced chronic obstructive pulmonary disease to identify and express their support needs to health care professionals: A qualitative study to develop a tool. Palliat Med. 2019;33(6):663-75.

44. Hahn‐Goldberg S, Okrainec K, Huynh T, Zahr N, Abrams H. Co‐creating patient‐oriented discharge instructions with patients, caregivers, and healthcare providers. J Hosp Med. 2015;10(12):804-7.

45. Hahn-Goldberg S, Damba C, Solomon F, Okrainec K, Abrams H, Huynh T. Using co-design methods to create a patient-oriented discharge summary. J Clin Outcomes Manag. 2016;23(7):321-8.

46. Han N, Han SH, Chu H, Kim J, Rhew KY, Yoon JH, et al. Service design oriented multidisciplinary collaborative team care service model development for resolving drug related problems. PLoS One. 2018;13(9).

47. Hickman IJ, Coran D, Wallen MP, Kelly J, Barnett A, Gallegos D, et al. 'Back to Life'-Using knowledge exchange processes to enhance lifestyle interventions for liver transplant recipients: A qualitative study. Nutr Diet. 2019;76(4):399-406.

48. Hobson EV, Baird WO, Partridge R, Cooper CL, Mawson S, Quinn A, et al. The TiM system: developing a novel telehealth service to improve access to specialist care in motor neurone disease using user-centered design. Amyotrophic Lateral Sclerosis and Frontotemporal Degeneration. 2018:1-11.

49. Holloway M. Traversing the network: a user‐led Care Pathway approach to the management of Parkinson's disease in the community. Health Soc Care Community. 2006;14(1):63-73.

50. Irving A, Turner J, Marsh M, Broadway-Parkinson A, Fall D, Coster J, et al. A coproduced patient and public event: An approach to developing and prioritizing ambulance performance measures. Health Expect. 2018;21(1):230-8.

51. Isenberg SR, Crossnohere NL, Patel MI, Conca-Cheng A, Bridges JFP, Swoboda SM, et al. An advance care plan decision support video before major surgery: a patient- and family-centred approach. BMJ Support Palliat Care. 2018;8(2):229‐36.

52. Jackson AM. ‘Follow the Fish’: involving young people in primary care in Midlothian. Health Expect. 2003;6(4):342-51.

53. Jessup RL, Osborne RH, Buchbinder R, Beauchamp A. Using co-design to develop interventions to address health literacy needs in a hospitalised population. BMC Health Serv Res [Internet]. 2018; 18(989). https://doi.org/10.1186/s12913-018-3801-7.

54. Jones SP, Auton MF, Burton CR, Watkins CL. Engaging service users in the development of stroke services: an action research study. J Clin Nurs. 2008;17(10):1270-9.

55. Kennedy A, Rogers A, Blickem C, Daker-White G, Bowen R. Developing cartoons for long-term condition self-management information. BMC Health Serv Res [Internet]. 2014; 14(60). https://doi.org/10.1186/1472-6963-14-60.

56. Kenyon SL, Johns N, Duggal S, Hewston R, Gale N. Improving the care pathway for women who request Caesarean section: an experience-based co-design study. BMC Pregnancy Childbirth [Internet]. 2016; 16(348). https://doi.org/10.1186/s12884-016-1134-2.

57. Kidd S, Kenny A, McKinstry C. Exploring the meaning of recovery-oriented care: An action-research study. Int J Ment Health Nurs. 2015;24(1):38-48.

58. Kilander H, Brynhildsen J, Alehagen SW, Fagerkrantz A, Thor J. Collaboratively seeking to improve contraceptive counselling at the time of an abortion: a case study of quality improvement efforts in Sweden. BMJ Sex Reprod Health. 2019;45(3):190-9.

59. Kildea S, Hickey S, Nelson C, Currie J, Carson A, Reynolds M, et al. Birthing on Country (in Our Community): a case study of engaging stakeholders and developing a best-practice Indigenous maternity service in an urban setting. Aust Health Rev. 2018;42(2):230-8.

60. Knight JA. Change management in cancer care: a one-stop gynaecology clinic. Br J Nurs. 2007;16(18):1122-6.

61. Kohler G, Sampalli T, Ryer A, Porter J, Wood L, Bedford L, et al. Bringing value-based perspectives to care: including patient and family members in decision-making processes. Int J Health Policy Manag. 2017;6(11):661-8.

62. Krist AH, Peele E, Woolf SH, Rothemich SF, Loomis JF, Longo DR, et al. Designing a patient-centered personal health record to promote preventive care. BMC Med Inform Decis Mak [Internet]. 2011; 11(73). https://doi.org/10.1186/1472-6947-11-73.

63. Larkin M, Boden ZV, Newton E. On the brink of genuinely collaborative care experience-based co-design in mental health. Qual Health Res. 2015;25(11):1463-76.

64. Latif A, Carter T, Rychwalska-Brown L, Wharrad H, Manning J. Co-producing a digital educational programme for registered children’s nurses to improve care of children and young people admitted with self-harm. J Child Health Care. 2017;21(2):191-200.

65. Lo C, Zimbudzi E, Teede H, Cass A, Fulcher G, Gallagher M, et al. Models of care for co‐morbid diabetes and chronic kidney disease. Nephrology. 2018;23(8):711-7.

66. Locock L, Robert G, Boaz A, Vougioukalou S, Shuldham C, Fielden J, et al. Using a national archive of patient experience narratives to promote local patient-centered quality improvement: an ethnographic process evaluation of ‘accelerated’experience-based co-design. J Health Serv Res Policy. 2014;19(4):200-7.

67. Lopatina E, Miller JL, Teare SR, Marlett NJ, Patel J, Barber CEH, et al. The voice of patients in system redesign: A case study of redesigning a centralized system for intake of referrals from primary care to rheumatologists for patients with suspected rheumatoid arthritis. Health Expect. 2019;22(3):348-63.

68. Lyles CR, Altschuler A, Chawla N, Kowalski C, McQuillan D, Bayliss E, et al. User-centered design of a tablet waiting room tool for complex patients to prioritize discussion topics for primary care visits. JMIR Mhealth Uhealth [Internet]. 2016; 4(3):e108. http://doi.org/10.2196/mhealth.6187.

69. Manning JC, Carter T, Latif A, Horsley A, Cooper J, Armstrong M, et al. ‘Our Care through Our Eyes’. Impact of a co-produced digital educational programme on nurses’ knowledge, confidence and attitudes in providing care for children and young people who have self-harmed: a mixed-methods study in the UK. BMJ Open [Internet]. 2017; 7(4):e014750. https://doi.org/10.1136/bmjopen-2016-014750.

70. Marshall M, Noble J, Davies H, Waterman H, Walshe K, Sheaff R, et al. Development of an information source for patients and the public about general practice services: an action research study. Health Expect. 2006;9(3):265-74.

71. McClelland GT, Fitzgerald M. A participatory mobile application (app) development project with mental health service users and clinicians. Health Educ J. 2018;77(7):815-27.

72. McWilliams A, Reeves K, Shade L, Burton E, Tapp H, Courtlandt C, et al. Patient and Family Engagement in the Design of a Mobile Health Solution for Pediatric Asthma: Development and Feasibility Study. JMIR Mhealth Uhealth. 2018;6(3):e68.

73. Meldrum J, Pringle A. Sex, lives and videotape. The journal of the Royal Society for the Promotion of Health. 2006;126(4):172-7.

74. Melnick ER, Hess EP, Guo G, Breslin M, Lopez K, Pavlo AJ, et al. Patient-centered decision support: formative usability evaluation of integrated clinical decision support with a patient decision aid for minor head injury in the emergency department. J Med Internet Res. 2017;19(5):1-12.

75. Olding M, Hayashi K, Pearce L, Bingham B, Buchholz M, Gregg D, et al. Developing a patient-reported experience questionnaire with and for people who use drugs: A community engagement process in Vancouver's Downtown Eastside. Int J Drug Policy. 2018;59:16-23.

76. Outlaw P, Tripathi S, Baldwin J. Using patient experiences to develop services for chronic pain. Br J Pain. 2018;12(2):122-31.

77. Owens C, Farrand P, Darvill R, Emmens T, Hewis E, Aitken P. Involving service users in intervention design: a participatory approach to developing a text-messaging intervention to reduce repetition of self-harm. Health Expect. 2011;14(3):285-95.

78. Pilgrim D, Waldron L. User involvement in mental health service development: how far can it go? J Ment Health. 1998;7(1):95-104.

79. Piper D, Iedema R, Gray J, Verma R, Holmes L, Manning N. Utilizing experience-based co-design to improve the experience of patients accessing emergency departments in New South Wales public hospitals: An evaluation study. Health Serv Manage Res. 2012;25(4):162-72.

80. Powell J, Lovelock R, Bray J, Philp I. Involving consumers in assessing service quality: benefits of using a qualitative approach. Qual Health Care. 1994;3(4):199-202.

81. Probst MA, Hess EP, Breslin M, Frosch DL, Sun BC, Langan M-N, et al. Development of a Patient Decision Aid for Syncope in the Emergency Department: the SynDA Tool. Acad Emerg Med. 2018;25(4):425-33.

82. Reaume-Zimmer P, Chandrasena R, Malla A, Joober R, Boksa P, Shah JL, et al. Transforming youth mental health care in a semi-urban and rural region of Canada: A service description of ACCESS Open Minds Chatham-Kent. Early Interv Psychiatry. 2019;13 Suppl 1:48-55.

83. Robinson LJ, Stephens NM, Wilson S, Graham L, Hackett KL. Conceptualizing the key components of rehabilitation following major musculoskeletal trauma: A mixed methods service evaluation. J Eval Clin Pract. 2019:1-12.

84. Romm KL, Gardsjord ES, Gjermundsen K, Ulloa MA, Berentzen LC, Melle I. Designing easy access to care for first-episode psychosis in complex organizations. Early Interv Psychiatry. 2019;13(5):1276-82.

85. Ruland CM, Starren J, Vatne TM. Participatory design with children in the development of a support system for patient-centered care in pediatric oncology. J Biomed Inform. 2008;41(4):624-35.

86. Taylor J, Coates E, Wessels B, Mountain G, Hawley MS. Implementing solutions to improve and expand telehealth adoption: participatory action research in four community healthcare settings. BMC Health Serv Res [Internet]. 2015 December 01; 15(529). https://doi.org/10.1186/s12913-015-1195-3.

87. Thomson A, Rivas C, Giovannoni G. Multiple sclerosis outpatient future groups: improving the quality of participant interaction and ideation tools within service improvement activities. BMC Health Serv Res [Internet]. 2015; 15(105). https://doi.org/10.1186/s12913-015-0773-8.

88. Tsianakas V, Robert G, Maben J, Richardson A, Dale C, Wiseman T. Implementing patient-centred cancer care: using experience-based co-design to improve patient experience in breast and lung cancer services. Support Care Cancer. 2012;20(11):2639-47.

89. Tsimicalis A, Rennick J, Stinson J, May SL, Louli J, Choquette A, et al. Usability Testing of an Interactive Communication Tool to Help Children Express Their Cancer Symptoms. J Pediatr Oncol Nurs. 2018;35(5):320-31.

90. Valaitis R, Longaphy J, Ploeg J, Agarwal G, Oliver D, Nair K, et al. Health TAPESTRY: co-designing interprofessional primary care programs for older adults using the persona-scenario method. BMC Fam Pract. 2019;20(1):122.

91. Wärnestål P, Svedberg P, Lindberg S, Nygren JM. Effects of using child personas in the development of a digital peer support service for childhood cancer survivors. J Med Internet Res. 2017;19(5):e161.

92. Woods L, Cummings E, Duff J, Walker K. Conceptual Design and Iterative Development of a mHealth App by Clinicians, Patients and Their Families. Stud Health Technol Inform. 2018;252:170-5.

93. Yu CH, Ke C, Jovicic A, Hall S, Straus SE. Beyond pros and cons - developing a patient decision aid to cultivate dialog to build relationships: insights from a qualitative study and decision aid development. BMC Med Inform Decis Mak. 2019;19(1):186.

94. Hong QN, Pluye P, Fabregues S, Bartlett G, Boardman F, Cargo M, et al. Mixed Methods Appraisal Tool (MMAT), version 2018: Canadian Intellectual Property Office, Industry Canada.; 2018.

95. Wright D, Foster C, Amir Z, Elliott J, Wilson R. Critical appraisal guidelines for assessing the quality and impact of user involvement in research. Health Expect. 2010;13(4):359-68.
